# Supplementary material for: iCanClean Improves Independent Component Analysis of Mobile Brain Imaging with EEG
Source: Sensors (Basel). 2023 Jan 13;23(2):928. doi: 10.3390/s23020928 (PMC9863946; doi:10.3390/s23020928)
Supplement: Supplementary file 1 [file sensors-23-00928-s001.zip › Supplemental Component Figures.pdf]

**Young Adult without using iCanClean** all plots are listed in decreasing %brain according to ICLabel, those highlighted in blue were >50% brain and had <15% RV

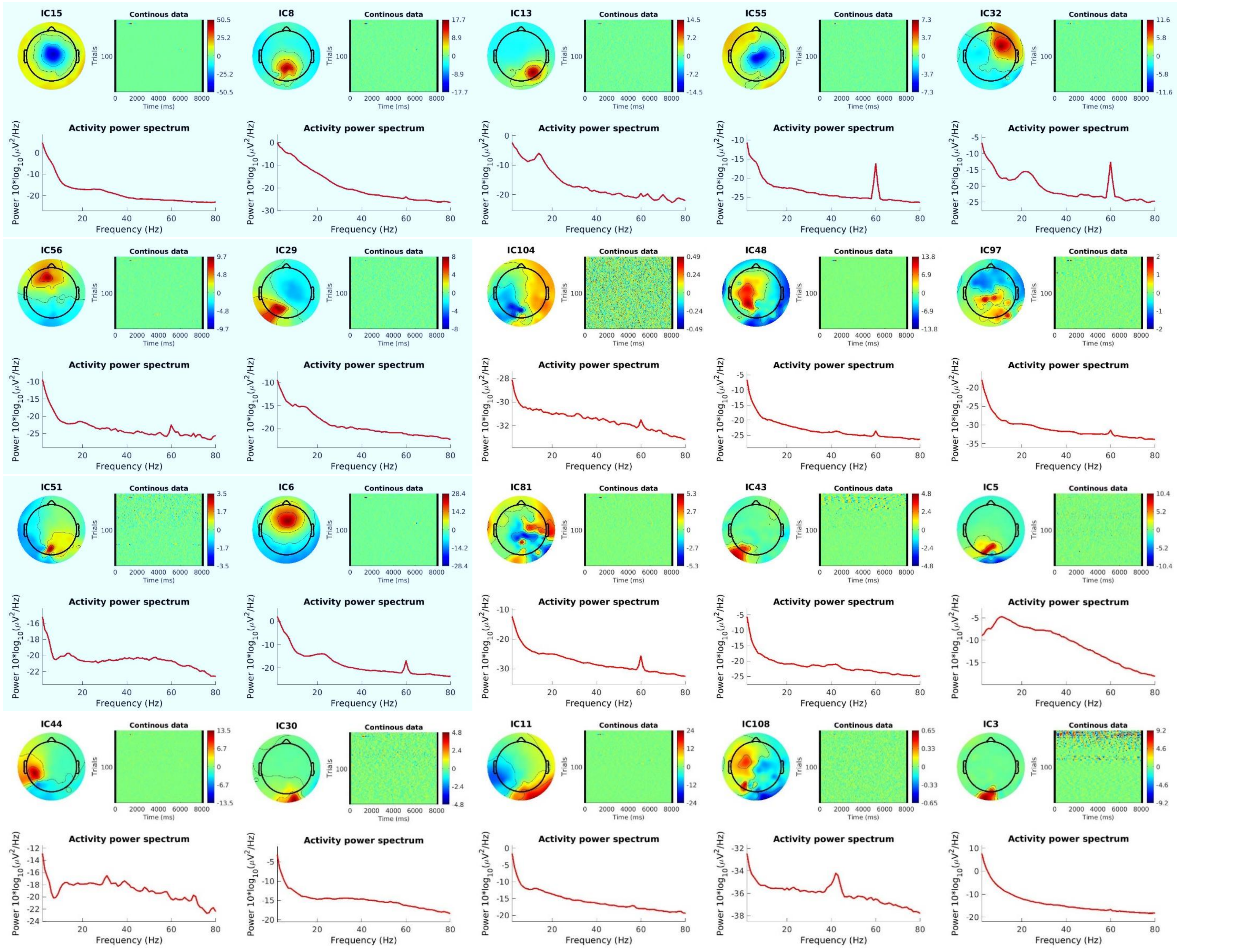

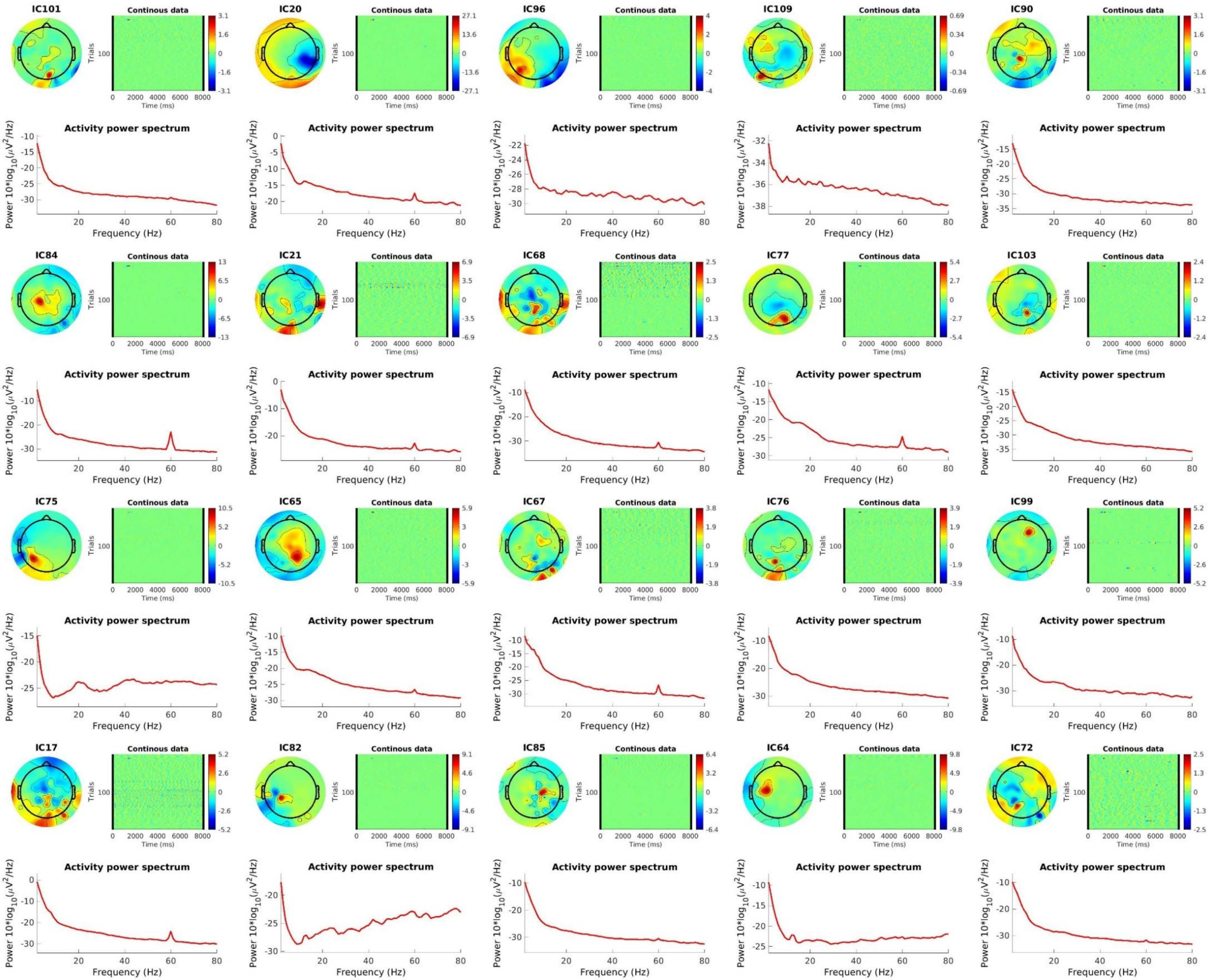
IC101
IC20
IC96
IC109
IC90
IC84
IC21
IC68
IC77
IC103
IC75
IC65
IC67
IC76
IC99
IC17
IC82
IC85
IC64
IC72
Continous data
Trials
Time (ms)
Activity power spectrum
Power 10*log10(μV²/Hz)
Frequency (Hz)

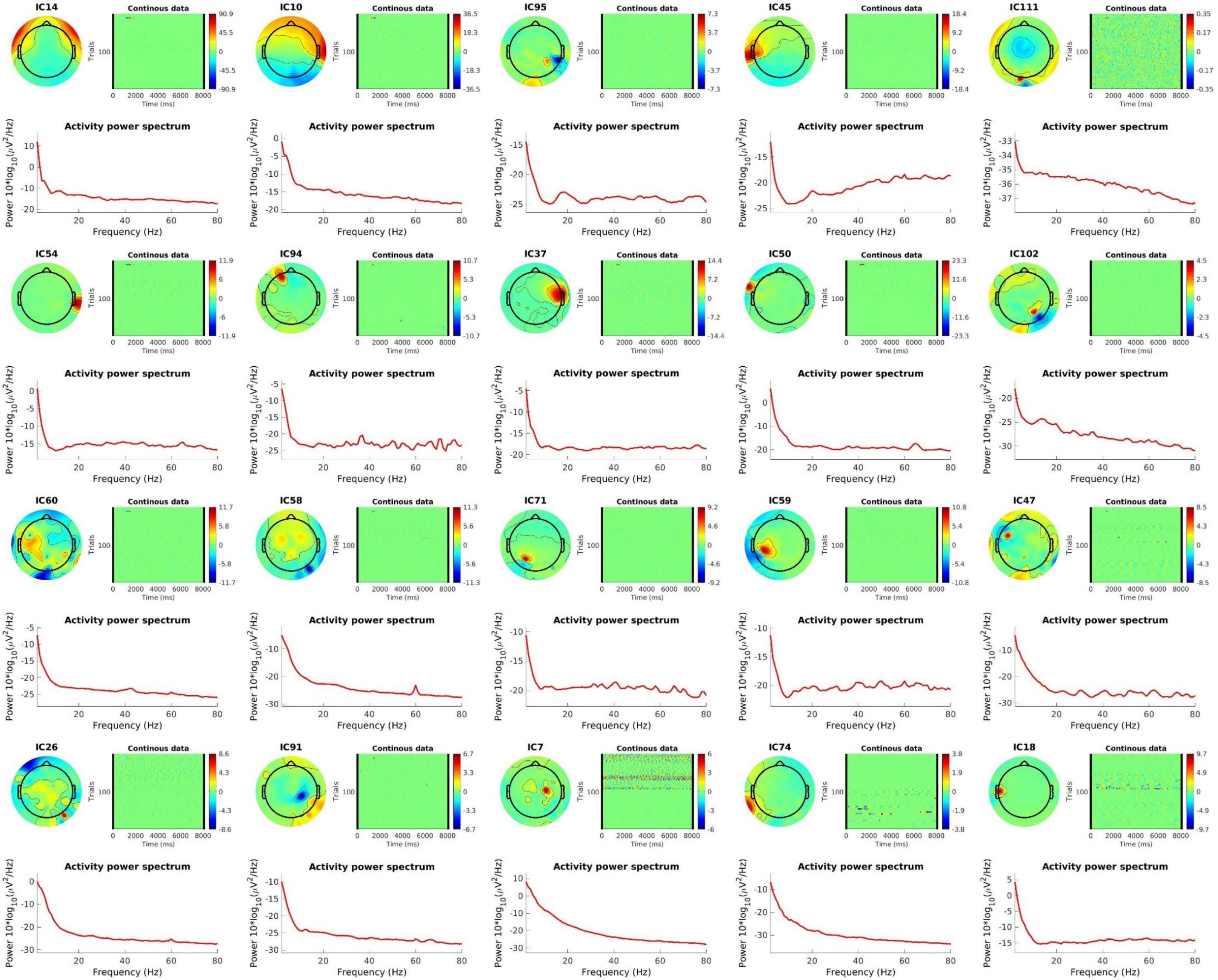

IC14
IC10
IC95
IC45
IC111
IC54
IC94
IC37
IC50
IC102
IC60
IC58
IC71
IC59
IC47
IC26
IC91
IC7
IC74
IC18
Continous data
Trials
Time (ms)
Activity power spectrum
Power 10*log10(μV2/Hz)
Frequency (Hz)

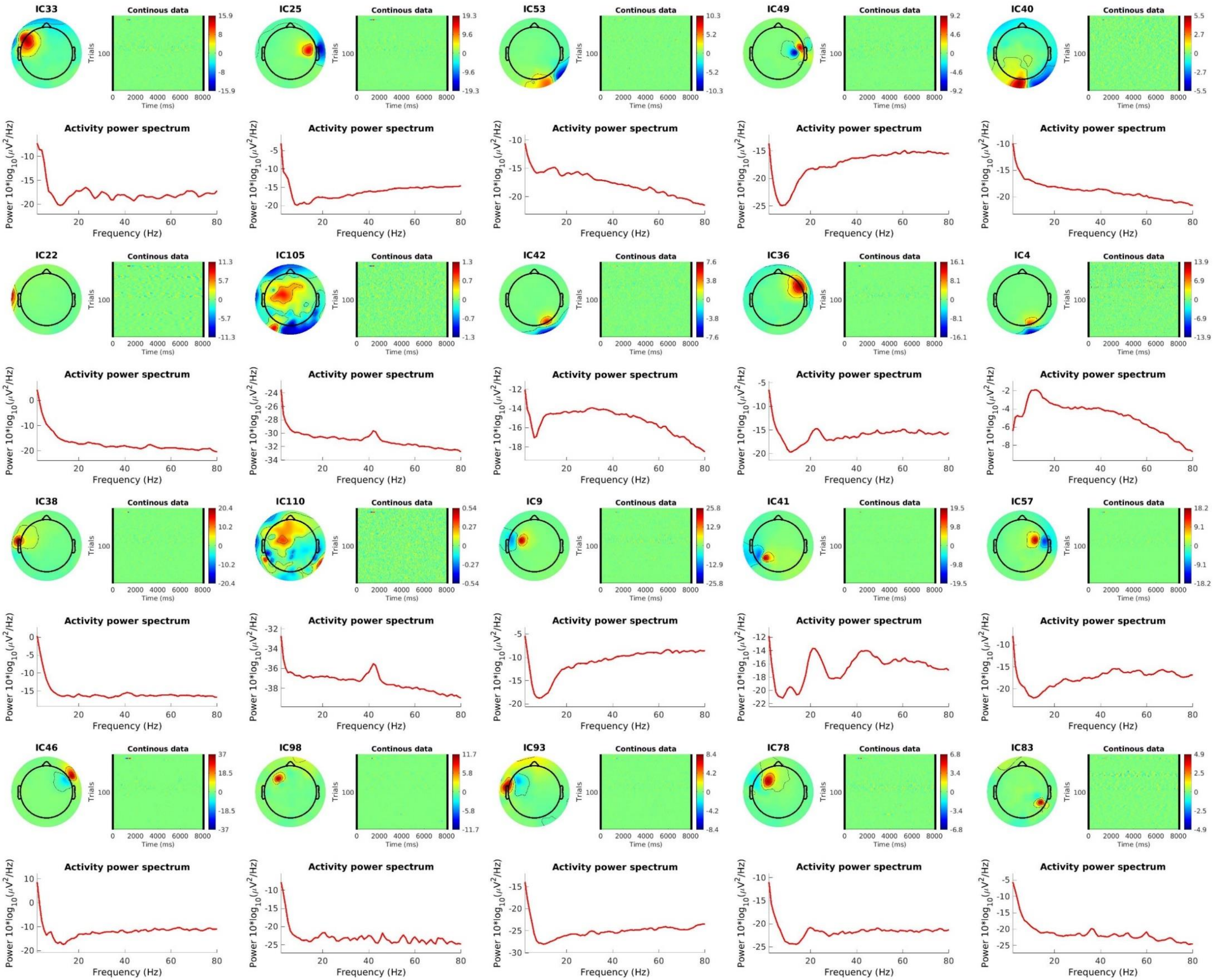
IC33
IC25
IC53
IC49
IC40
IC22
IC105
IC42
IC36
IC4
IC38
IC110
IC9
IC41
IC57
IC46
IC98
IC93
IC78
IC83
Continous data
Trials
Time (ms)
Activity power spectrum
Power 10*log10(μV2/Hz)
Frequency (Hz)

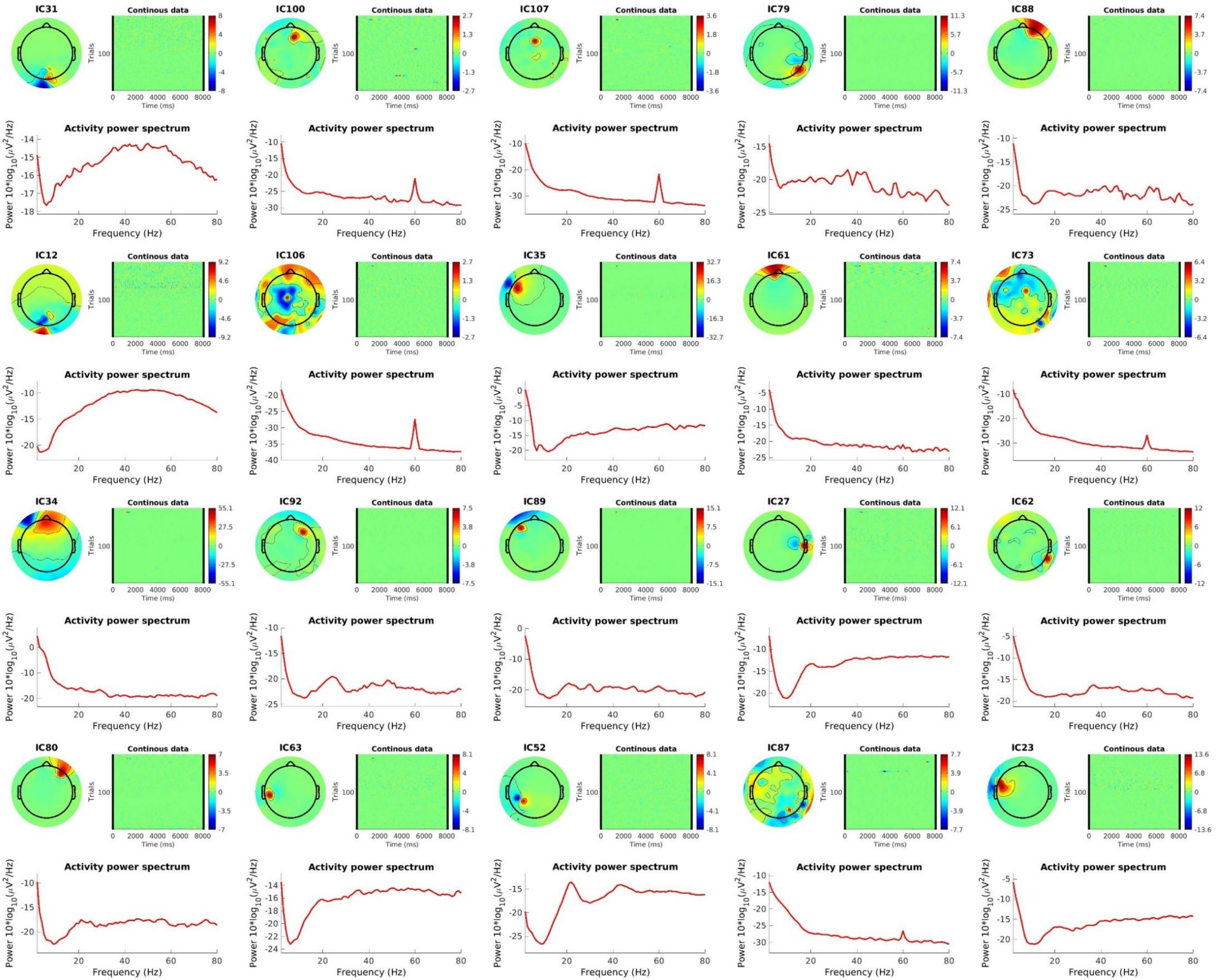
IC31
Continous data
Activity power spectrum
IC100
Continous data
Activity power spectrum
IC107
Continous data
Activity power spectrum
IC79
Continous data
Activity power spectrum
IC88
Continous data
Activity power spectrum
IC12
Continous data
Activity power spectrum
IC106
Continous data
Activity power spectrum
IC35
Continous data
Activity power spectrum
IC61
Continous data
Activity power spectrum
IC73
Continous data
Activity power spectrum
IC34
Continous data
Activity power spectrum
IC92
Continous data
Activity power spectrum
IC89
Continous data
Activity power spectrum
IC27
Continous data
Activity power spectrum
IC62
Continous data
Activity power spectrum
IC80
Continous data
Activity power spectrum
IC63
Continous data
Activity power spectrum
IC52
Continous data
Activity power spectrum
IC87
Continous data
Activity power spectrum
IC23
Continous data
Activity power spectrum
Trials
Time (ms)
Power 10*log$_{10}$($\mu$V$^2$/Hz)
Frequency (Hz)

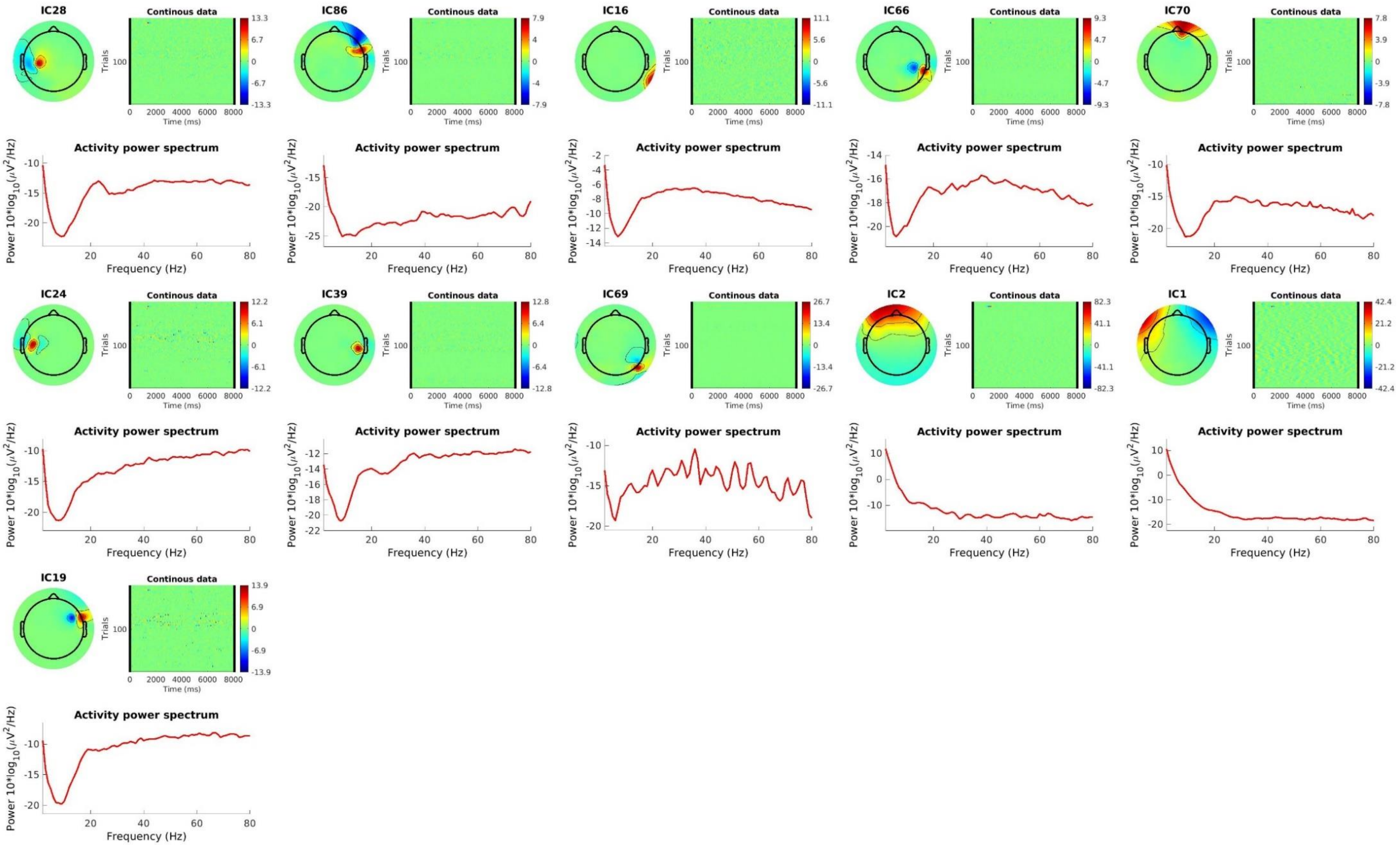
IC28
Continous data
Trials
Time (ms)
Activity power spectrum
Power 10*log10(μV²/Hz)
Frequency (Hz)
IC86
IC16
IC66
IC70
IC24
IC39
IC69
IC2
IC1
IC19

## Same Young Adult with iCanClean

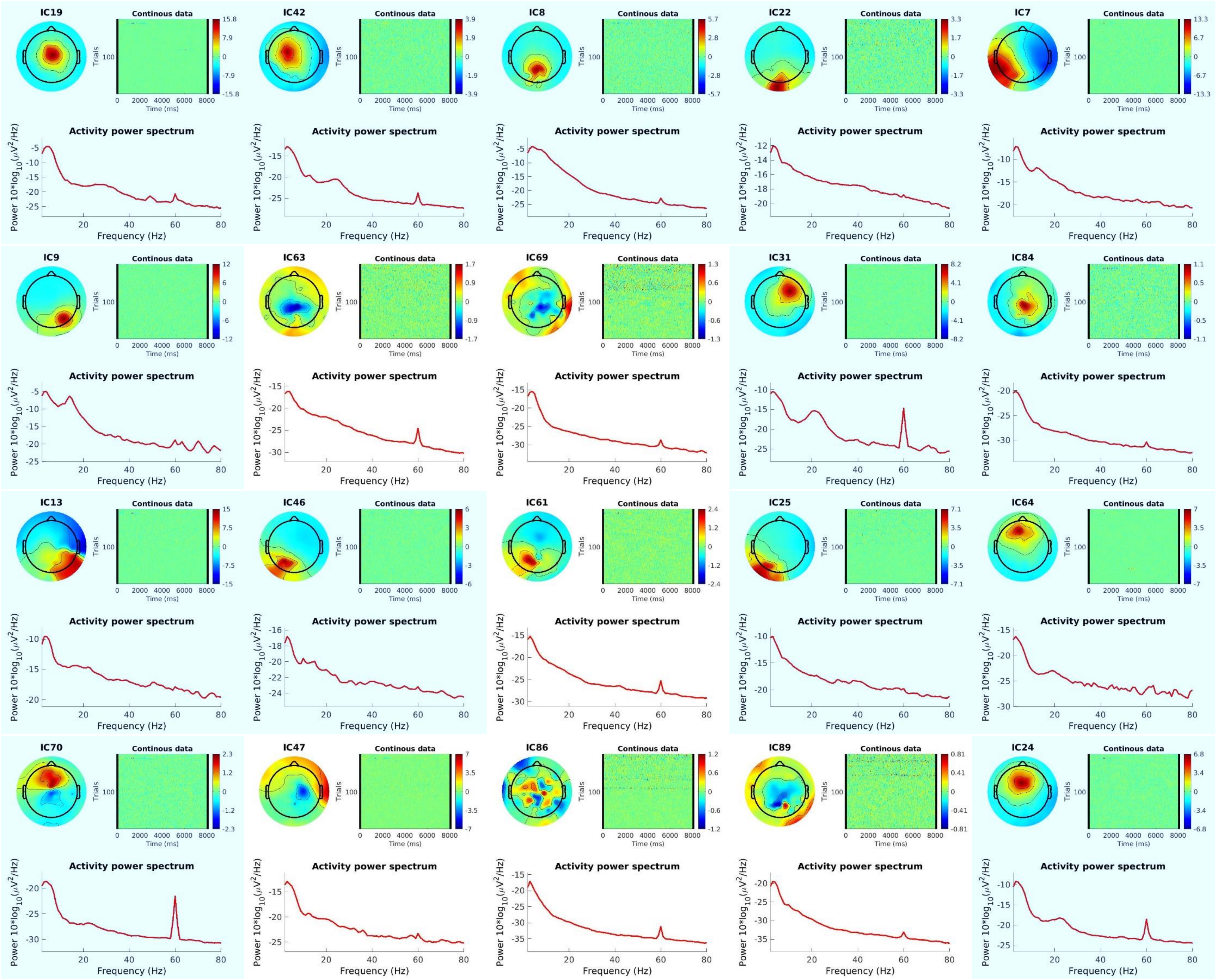

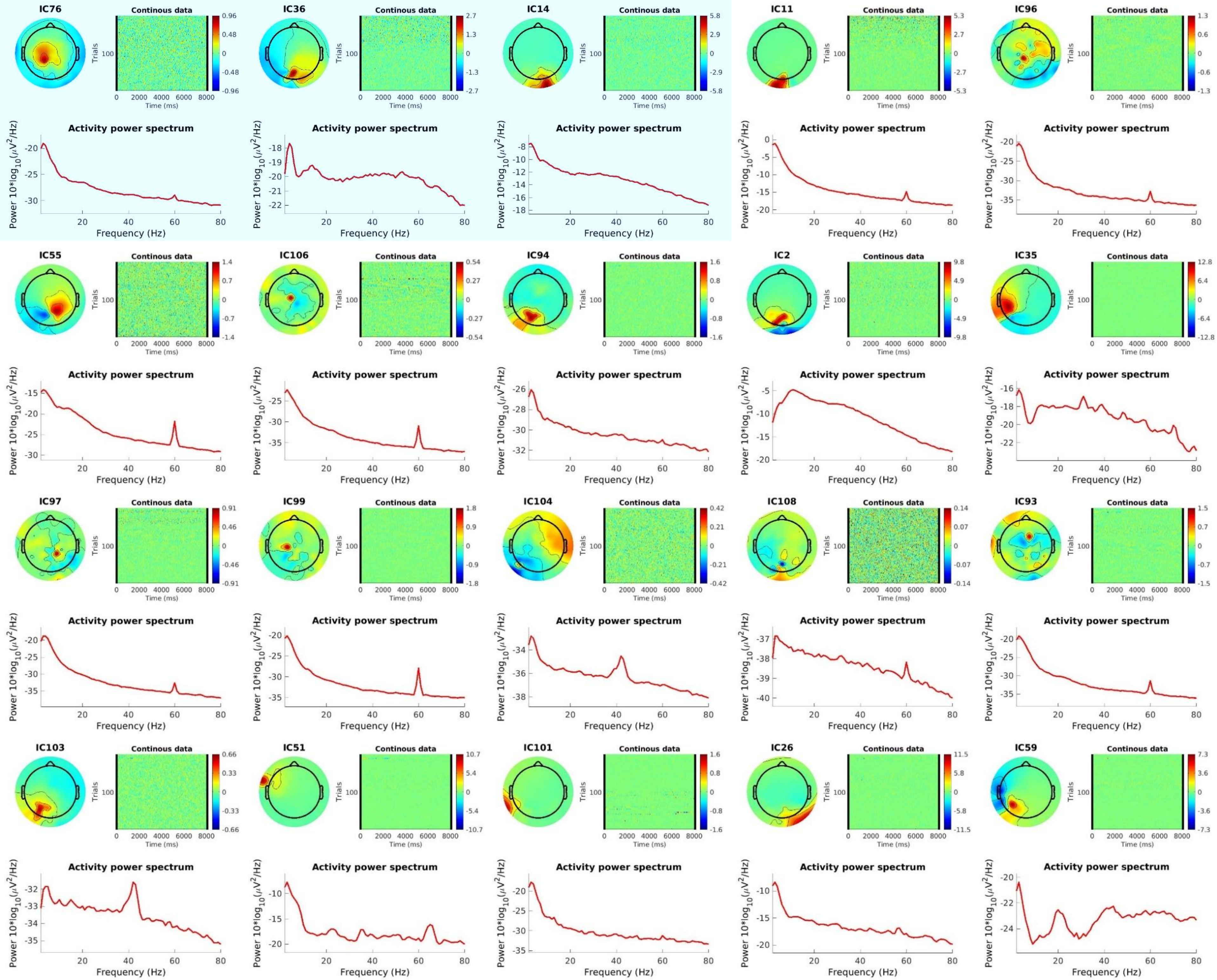

IC76
Continous data
Activity power spectrum
IC36
Continous data
Activity power spectrum
IC14
Continous data
Activity power spectrum
IC11
Continous data
Activity power spectrum
IC96
Continous data
Activity power spectrum
IC55
Continous data
Activity power spectrum
IC106
Continous data
Activity power spectrum
IC94
Continous data
Activity power spectrum
IC2
Continous data
Activity power spectrum
IC35
Continous data
Activity power spectrum
IC97
Continous data
Activity power spectrum
IC99
Continous data
Activity power spectrum
IC104
Continous data
Activity power spectrum
IC108
Continous data
Activity power spectrum
IC93
Continous data
Activity power spectrum
IC103
Continous data
Activity power spectrum
IC51
Continous data
Activity power spectrum
IC101
Continous data
Activity power spectrum
IC26
Continous data
Activity power spectrum
IC59
Continous data
Activity power spectrum
Trials
Time (ms)
Power 10*log10(μV2/Hz)
Frequency (Hz)

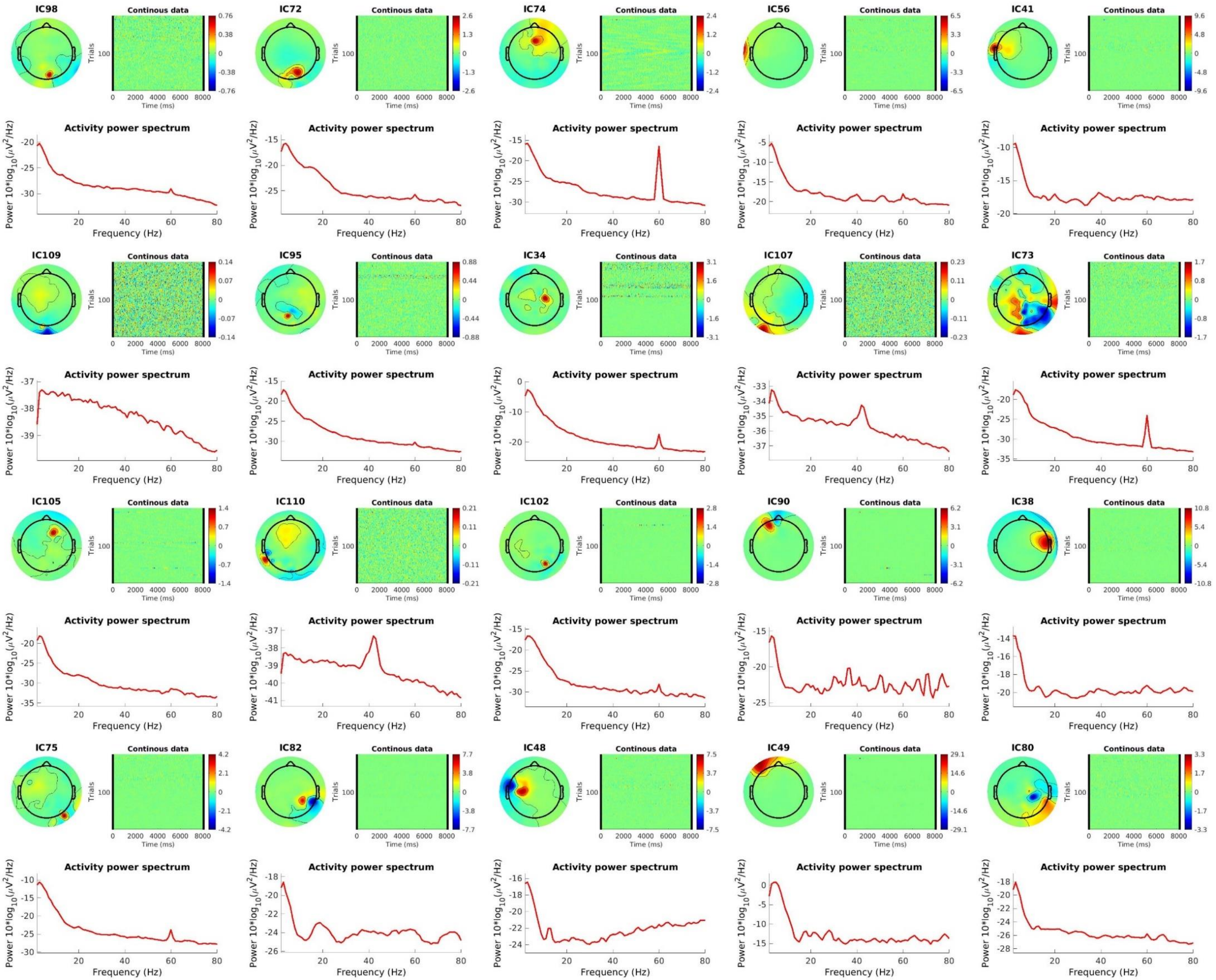
IC98
IC72
IC74
IC56
IC41
IC109
IC95
IC34
IC107
IC73
IC105
IC110
IC102
IC90
IC38
IC75
IC82
IC48
IC49
IC80
Continous data
Trials
Time (ms)
Activity power spectrum
Power 10*log$_{10}$($\mu$V$^2$/Hz)
Frequency (Hz)

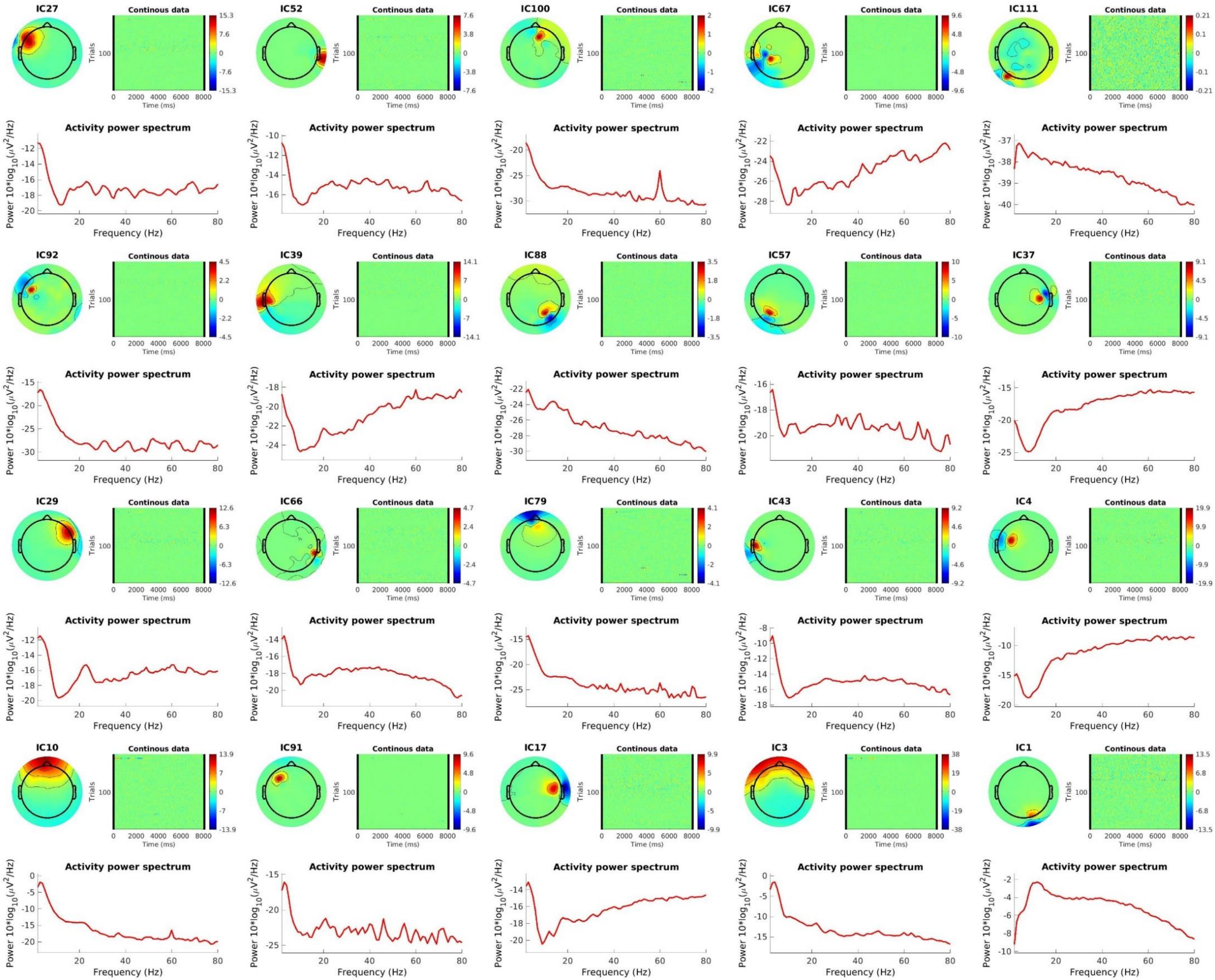
IC27
Continous data
Trials
Time (ms)
Activity power spectrum
Power 10*log10(μV2/Hz)
Frequency (Hz)
IC52
Continous data
Activity power spectrum
IC100
Continous data
Activity power spectrum
IC67
Continous data
Activity power spectrum
IC111
Continous data
Activity power spectrum
IC92
Continous data
Activity power spectrum
IC39
Continous data
Activity power spectrum
IC88
Continous data
Activity power spectrum
IC57
Continous data
Activity power spectrum
IC37
Continous data
Activity power spectrum
IC29
Continous data
Activity power spectrum
IC66
Continous data
Activity power spectrum
IC79
Continous data
Activity power spectrum
IC43
Continous data
Activity power spectrum
IC4
Continous data
Activity power spectrum
IC10
Continous data
Activity power spectrum
IC91
Continous data
Activity power spectrum
IC17
Continous data
Activity power spectrum
IC3
Continous data
Activity power spectrum
IC1
Continous data
Activity power spectrum

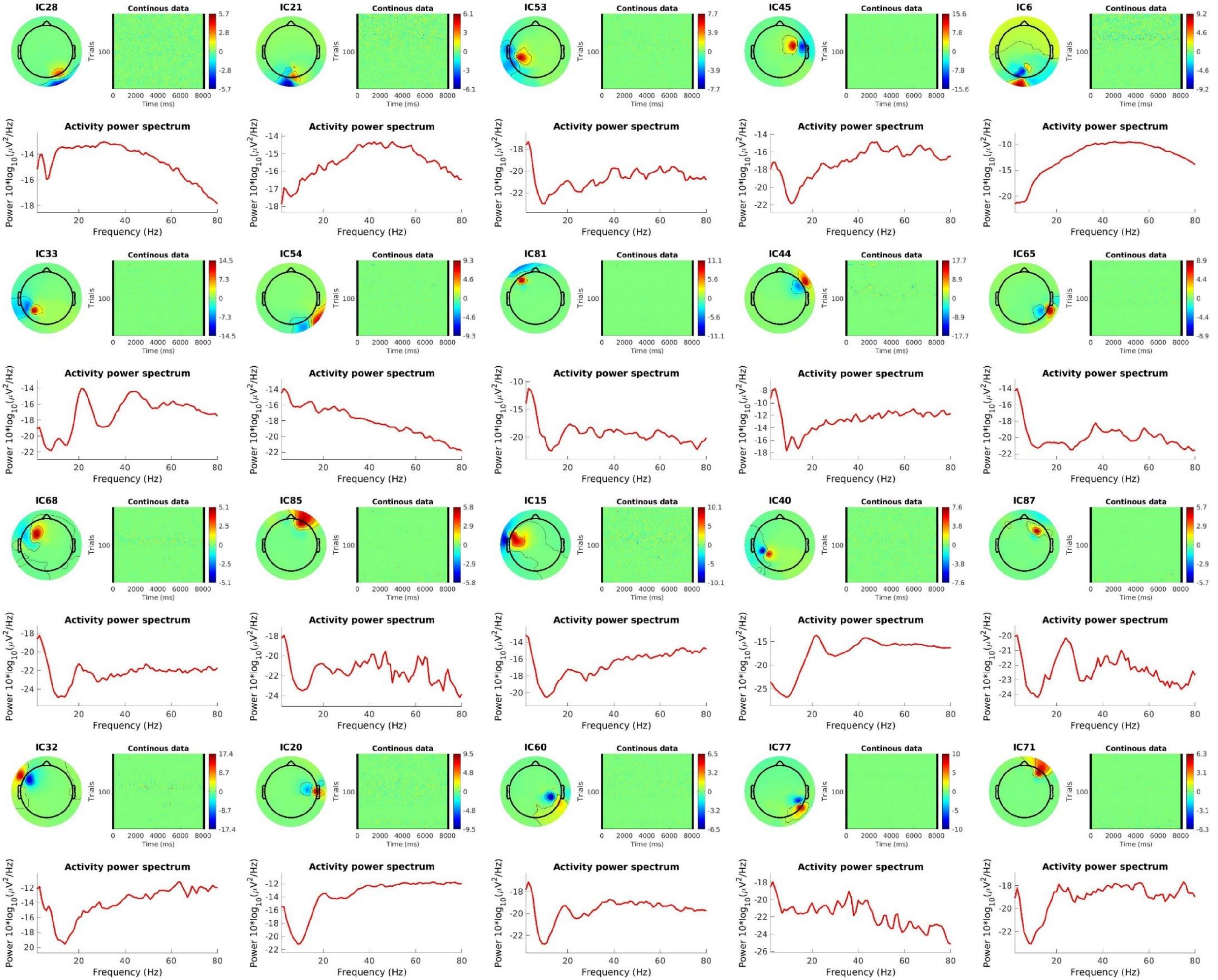
IC28
IC21
IC53
IC45
IC6
IC33
IC54
IC81
IC44
IC65
IC68
IC85
IC15
IC40
IC87
IC32
IC20
IC60
IC77
IC71
Continous data
Trials
Time (ms)
Activity power spectrum
Power 10*log$_{10}$($\mu$V$^2$/Hz)
Frequency (Hz)

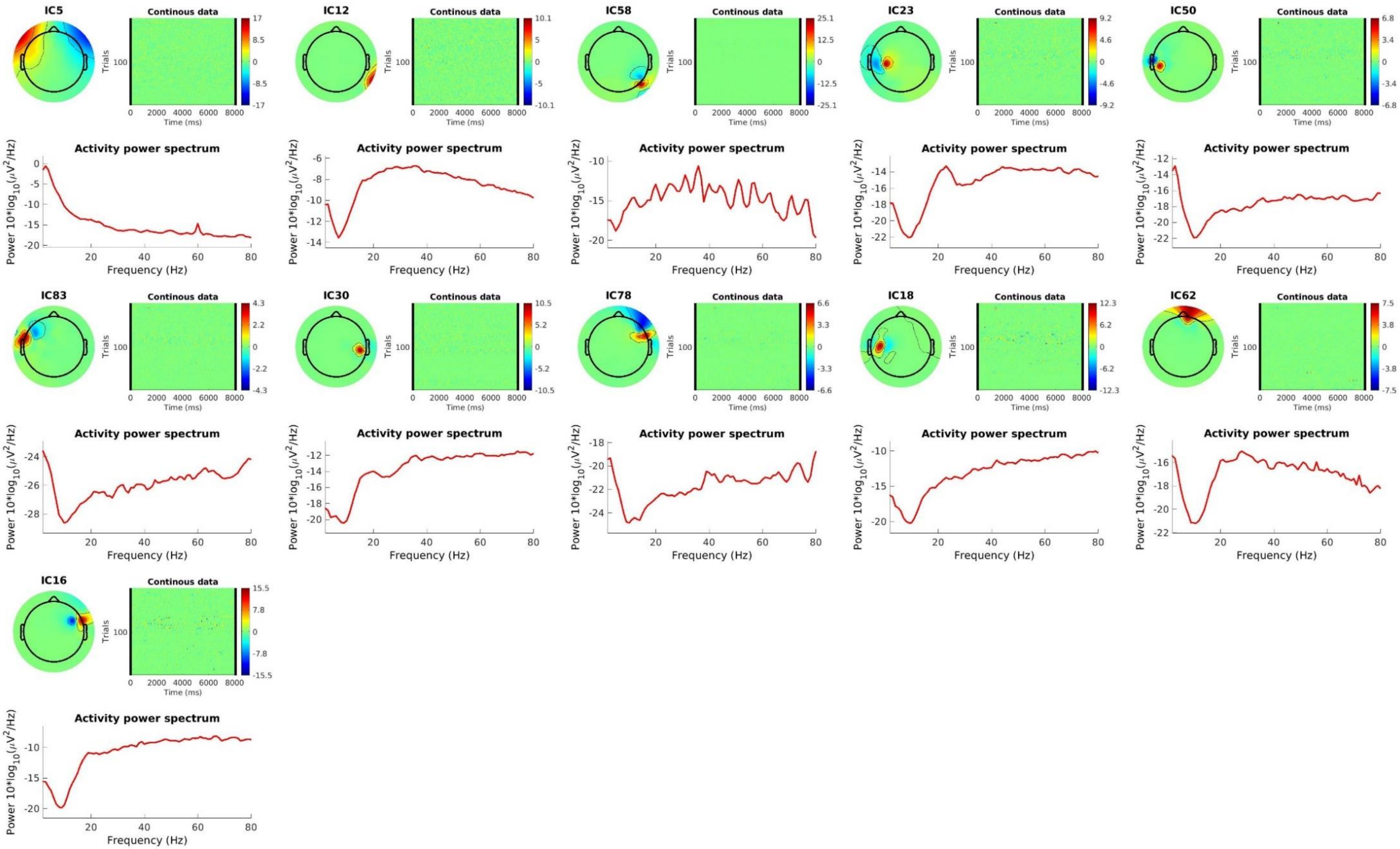

IC5
IC12
IC58
IC23
IC50
IC83
IC30
IC78
IC18
IC62
IC16
Continous data
Trials
Time (ms)
Activity power spectrum
Power 10*log10(μV2/Hz)
Frequency (Hz)

# High Functioning Older Adult Without iCanClean

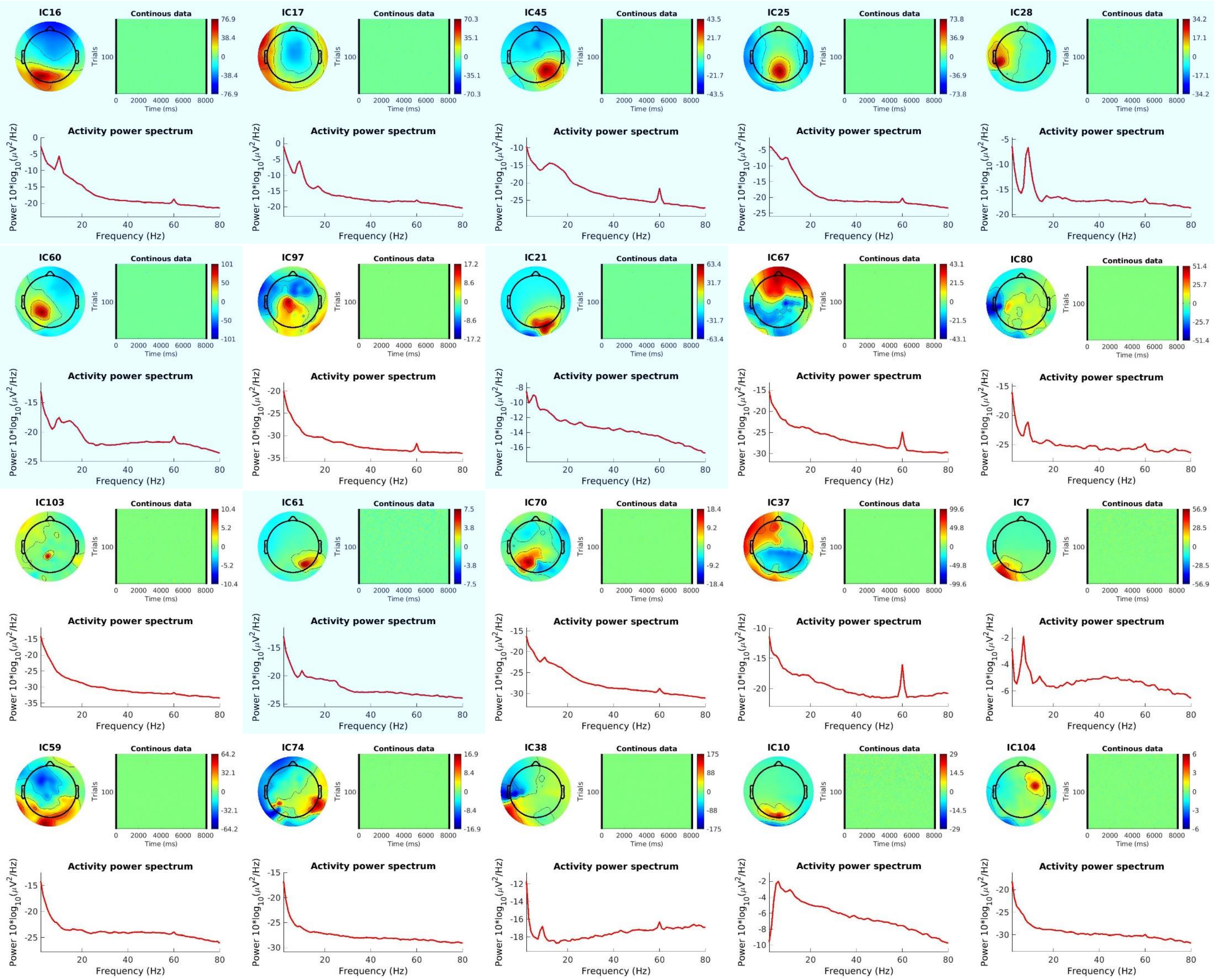

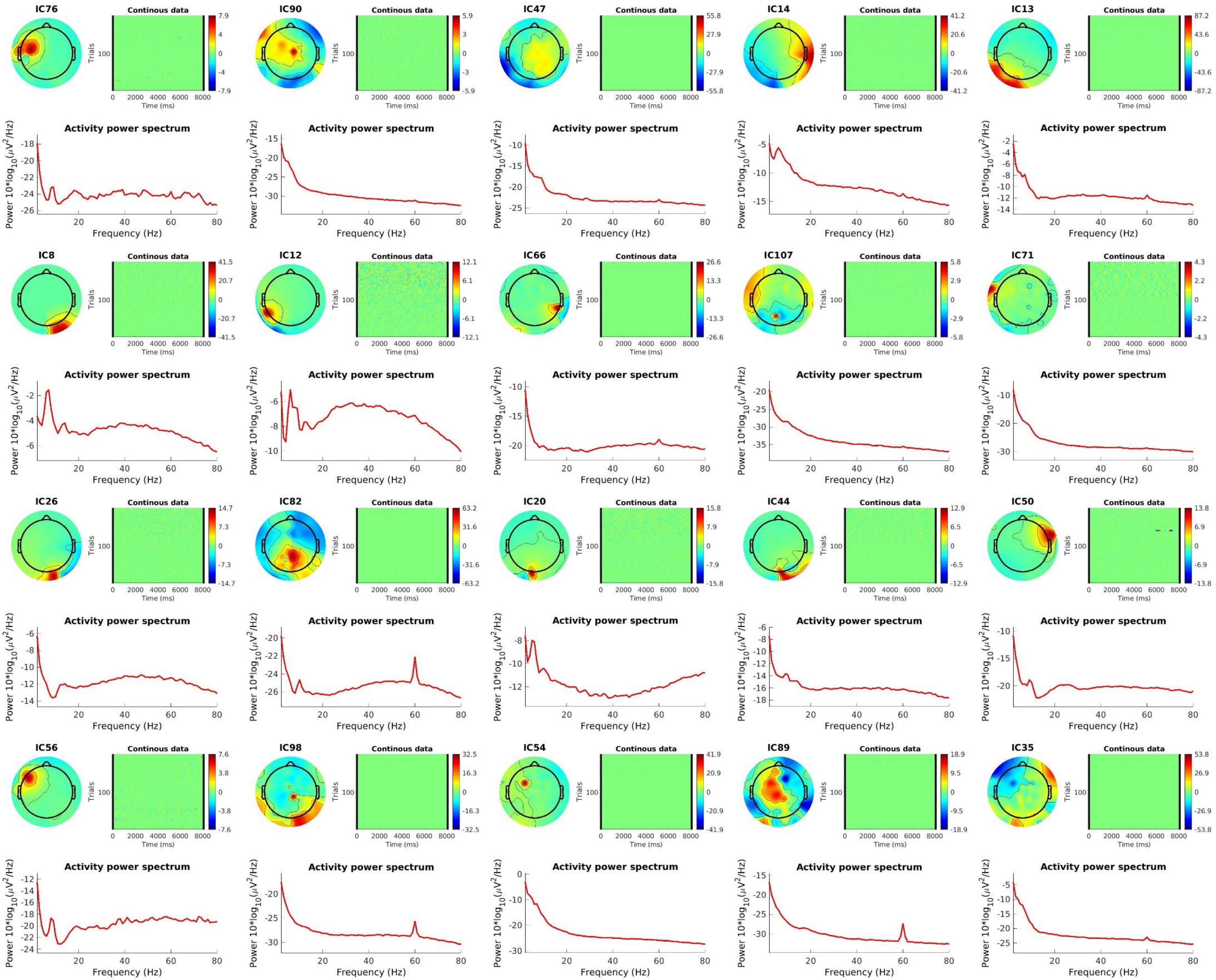
IC76
IC90
IC47
IC14
IC13
IC8
IC12
IC66
IC107
IC71
IC26
IC82
IC20
IC44
IC50
IC56
IC98
IC54
IC89
IC35
Continous data
Trials
Time (ms)
Activity power spectrum
Power 10*log10(μV2/Hz)
Frequency (Hz)

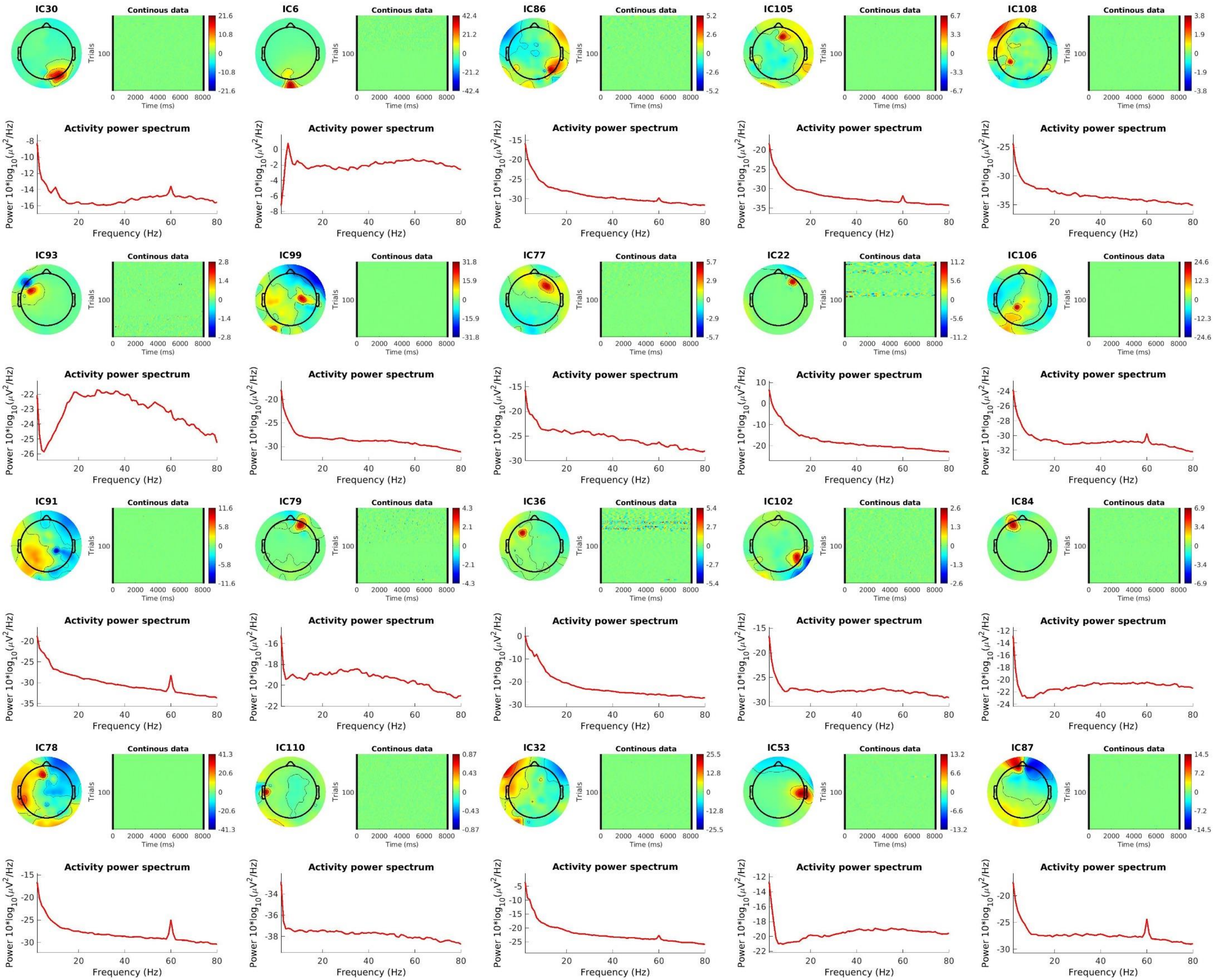
IC30
IC6
IC86
IC105
IC108
IC93
IC99
IC77
IC22
IC106
IC91
IC79
IC36
IC102
IC84
IC78
IC110
IC32
IC53
IC87
Continous data
Trials
Time (ms)
Activity power spectrum
Power 10*log$_{10}$($\mu$V$^2$/Hz)
Frequency (Hz)

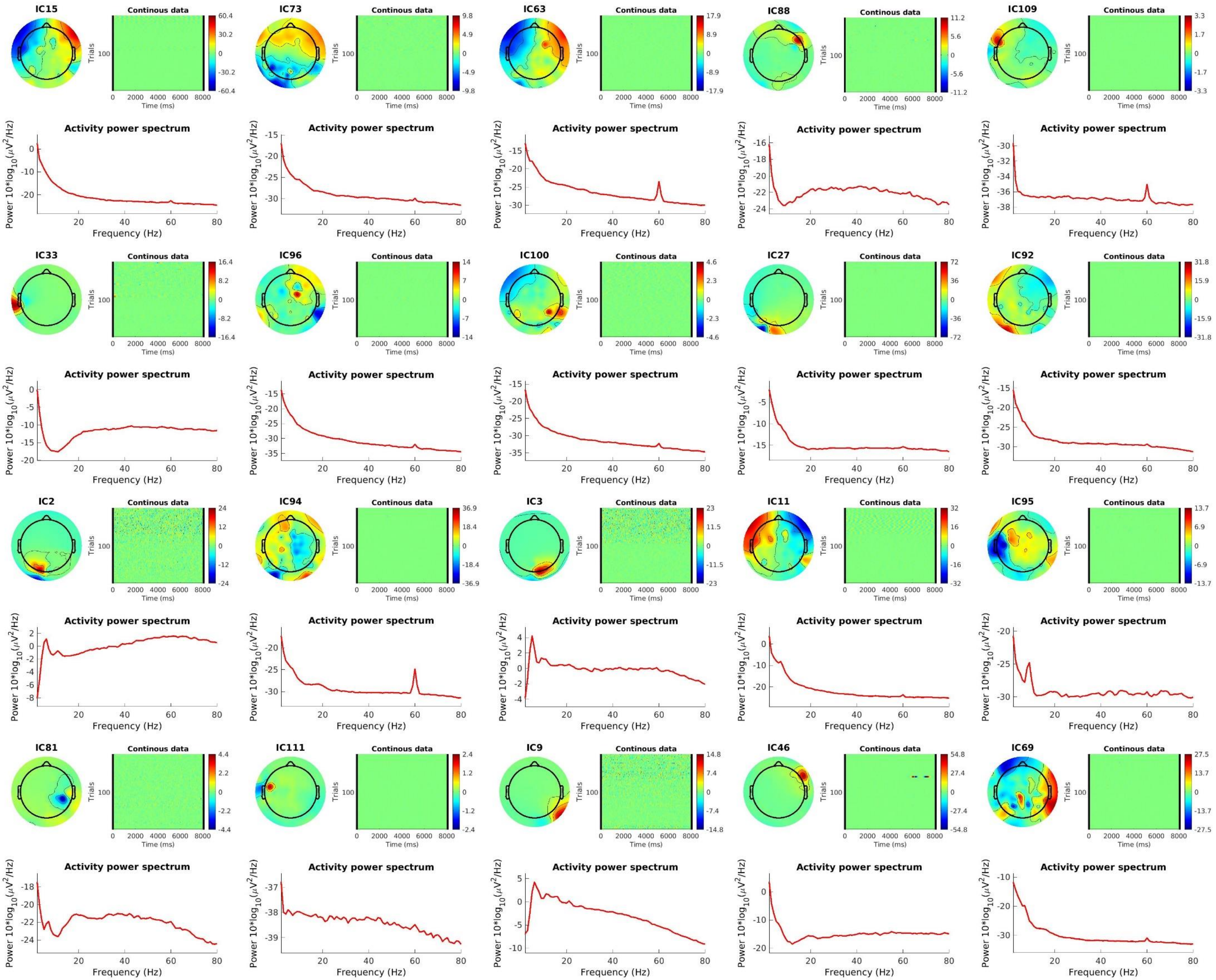
IC15
IC73
IC63
IC88
IC109
IC33
IC96
IC100
IC27
IC92
IC2
IC94
IC3
IC11
IC95
IC81
IC111
IC9
IC46
IC69
Continous data
Trials
Time (ms)
Activity power spectrum
Power 10*log$_{10}$($\mu V^2$/Hz)
Frequency (Hz)

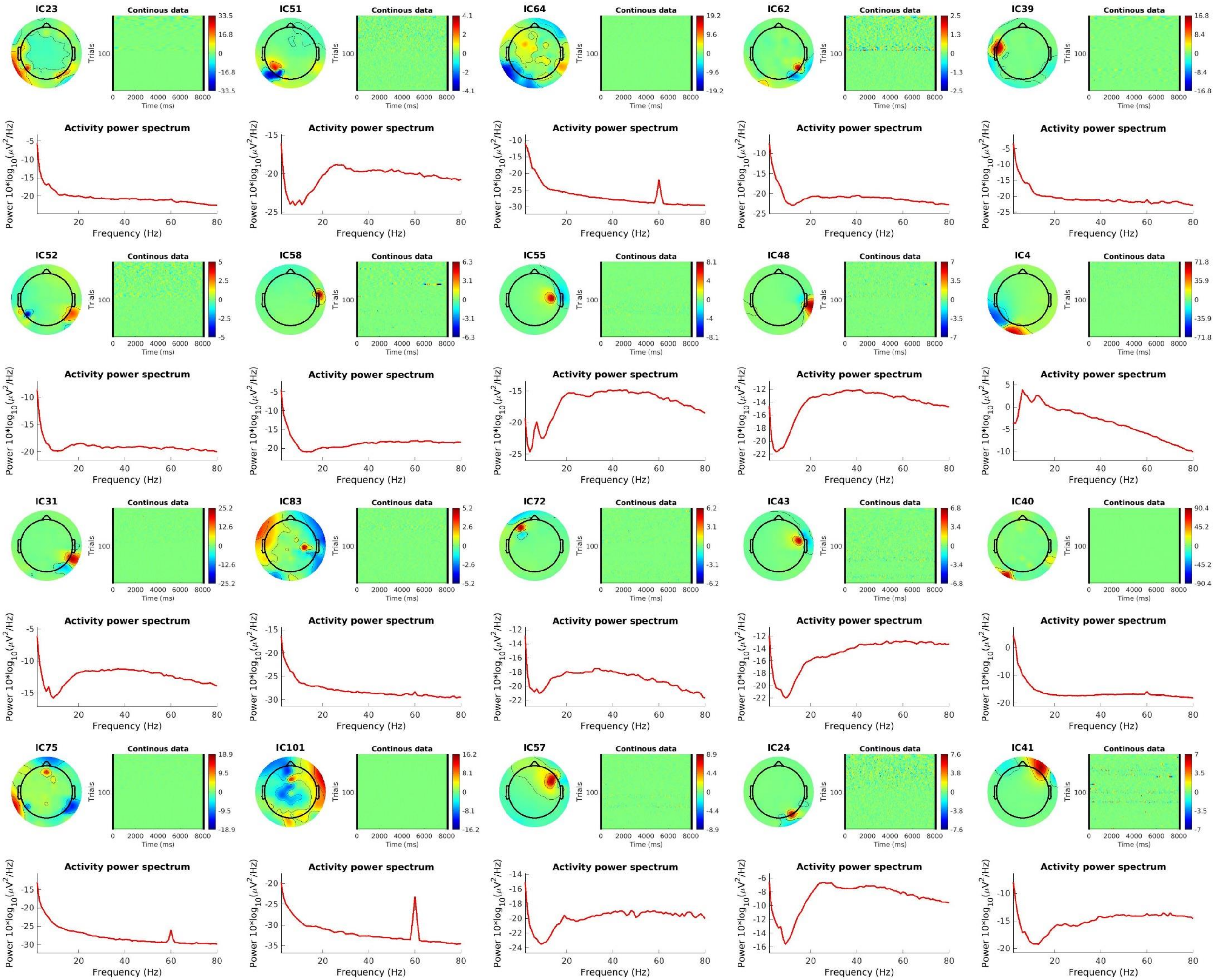

IC23
IC51
IC64
IC62
IC39
IC52
IC58
IC55
IC48
IC4
IC31
IC83
IC72
IC43
IC40
IC75
IC101
IC57
IC24
IC41
Continous data
Trials
Time (ms)
Activity power spectrum
Power 10*log10(μV2/Hz)
Frequency (Hz)

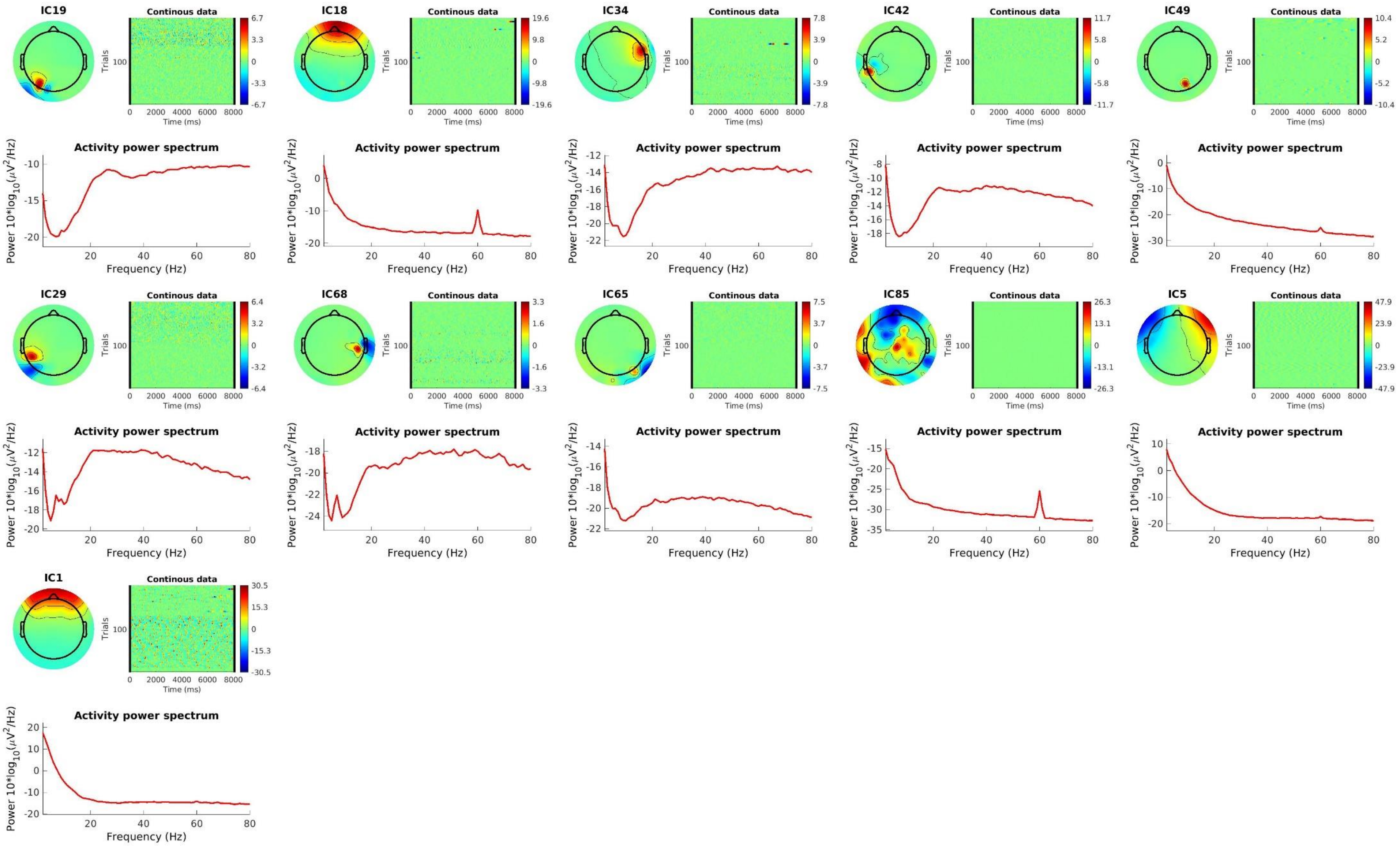
IC19
Continous data
Trials
Time (ms)
Activity power spectrum
Power 10*log10(μV2/Hz)
Frequency (Hz)
IC18
IC34
IC42
IC49
IC29
IC68
IC65
IC85
IC5
IC1

# High Functioning Older Adult With iCanClean

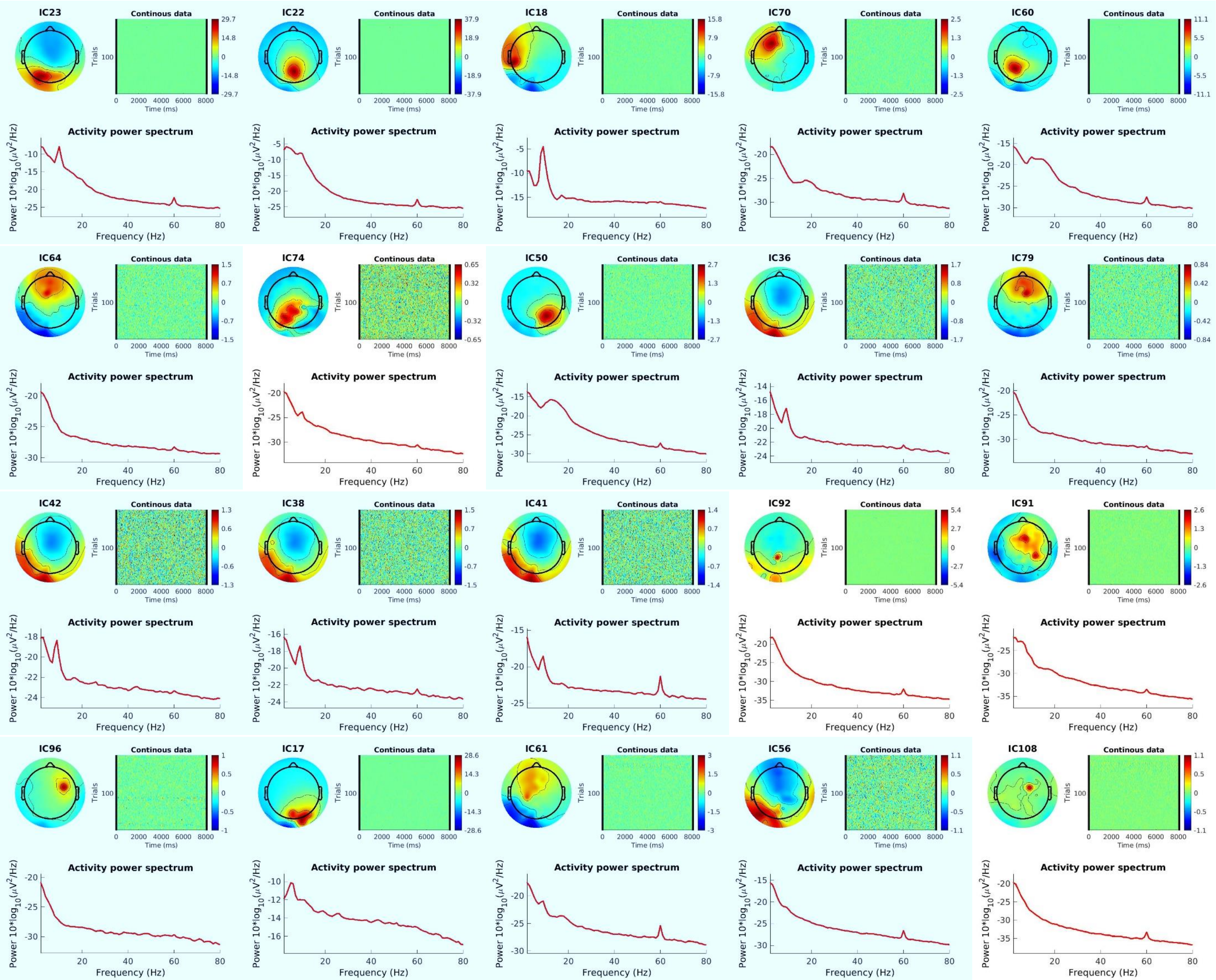

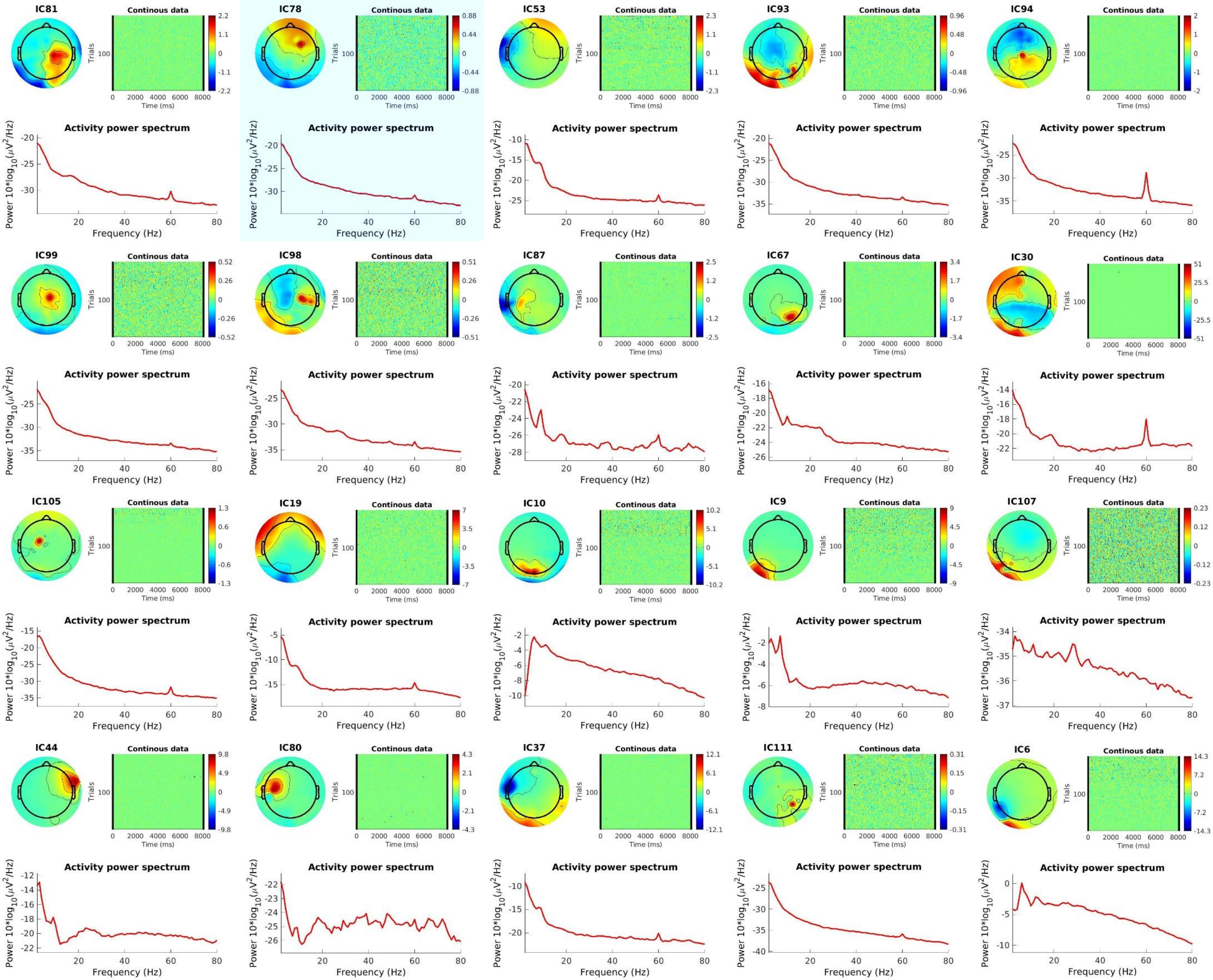

IC81
Continous data
2.2
1.1
0
-1.1
-2.2
Trials
100
0 2000 4000 6000 8000
Time (ms)
Activity power spectrum
Power 10*log10(μV2/Hz)
Frequency (Hz)
IC78
Continous data
0.88
0.44
0
-0.44
-0.88
IC53
Continous data
2.3
1.1
0
-1.1
-2.3
IC93
Continous data
0.96
0.48
0
-0.48
-0.96
IC94
Continous data
2
1
0
-1
-2
IC99
Continous data
0.52
0.26
0
-0.26
-0.52
IC98
Continous data
0.51
0.26
0
-0.26
-0.51
IC87
Continous data
2.5
1.2
0
-1.2
-2.5
IC67
Continous data
3.4
1.7
0
-1.7
-3.4
IC30
Continous data
51
25.5
0
-25.5
-51
IC105
Continous data
1.3
0.6
0
-0.6
-1.3
IC19
Continous data
7
3.5
0
-3.5
-7
IC10
Continous data
10.2
5.1
0
-5.1
-10.2
IC9
Continous data
9
4.5
0
-4.5
-9
IC107
Continous data
0.23
0.12
0
-0.12
-0.23
IC44
Continous data
9.8
4.9
0
-4.9
-9.8
IC80
Continous data
4.3
2.1
0
-2.1
-4.3
IC37
Continous data
12.1
6.1
0
-6.1
-12.1
IC111
Continous data
0.31
0.15
0
-0.15
-0.31
IC6
Continous data
14.3
7.2
0
-7.2
-14.3

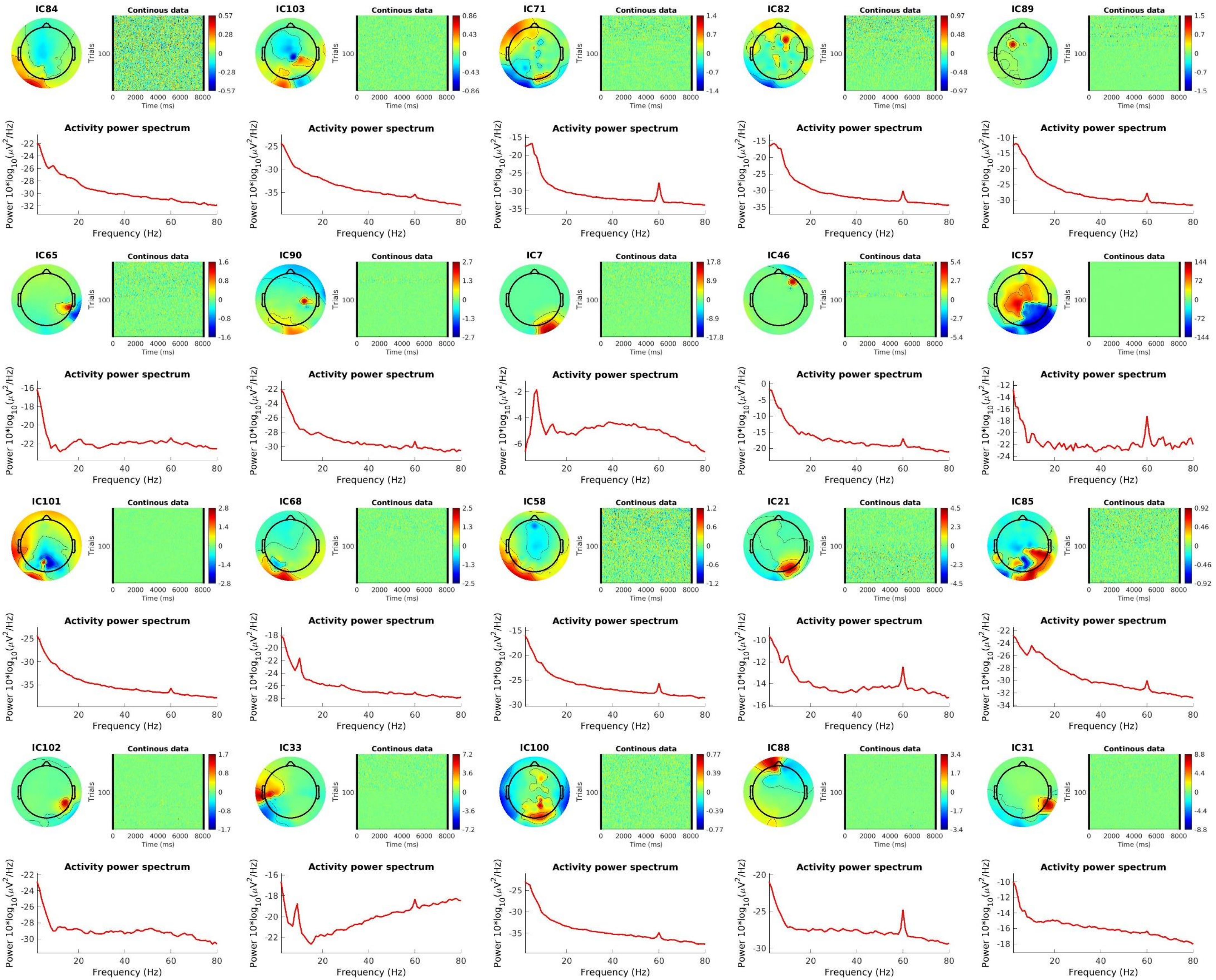
IC84
IC103
IC71
IC82
IC89
IC65
IC90
IC7
IC46
IC57
IC101
IC68
IC58
IC21
IC85
IC102
IC33
IC100
IC88
IC31
Continous data
Trials
Time (ms)
Activity power spectrum
Power 10*log10(μV2/Hz)
Frequency (Hz)

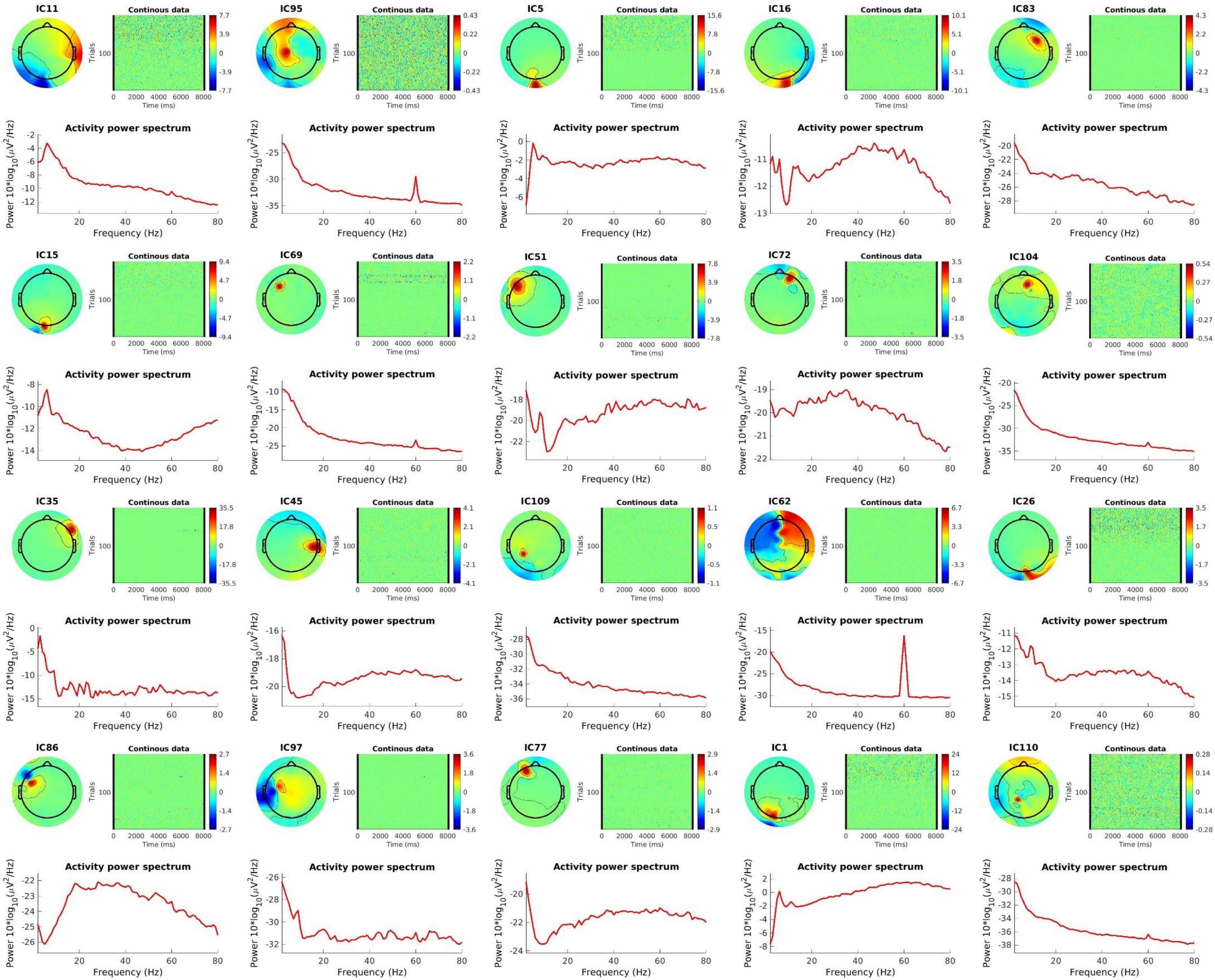
IC11
IC95
IC5
IC16
IC83
IC15
IC69
IC51
IC72
IC104
IC35
IC45
IC109
IC62
IC26
IC86
IC97
IC77
IC1
IC110
Continous data
Trials
Time (ms)
Activity power spectrum
Power 10*log10(μV2/Hz)
Frequency (Hz)

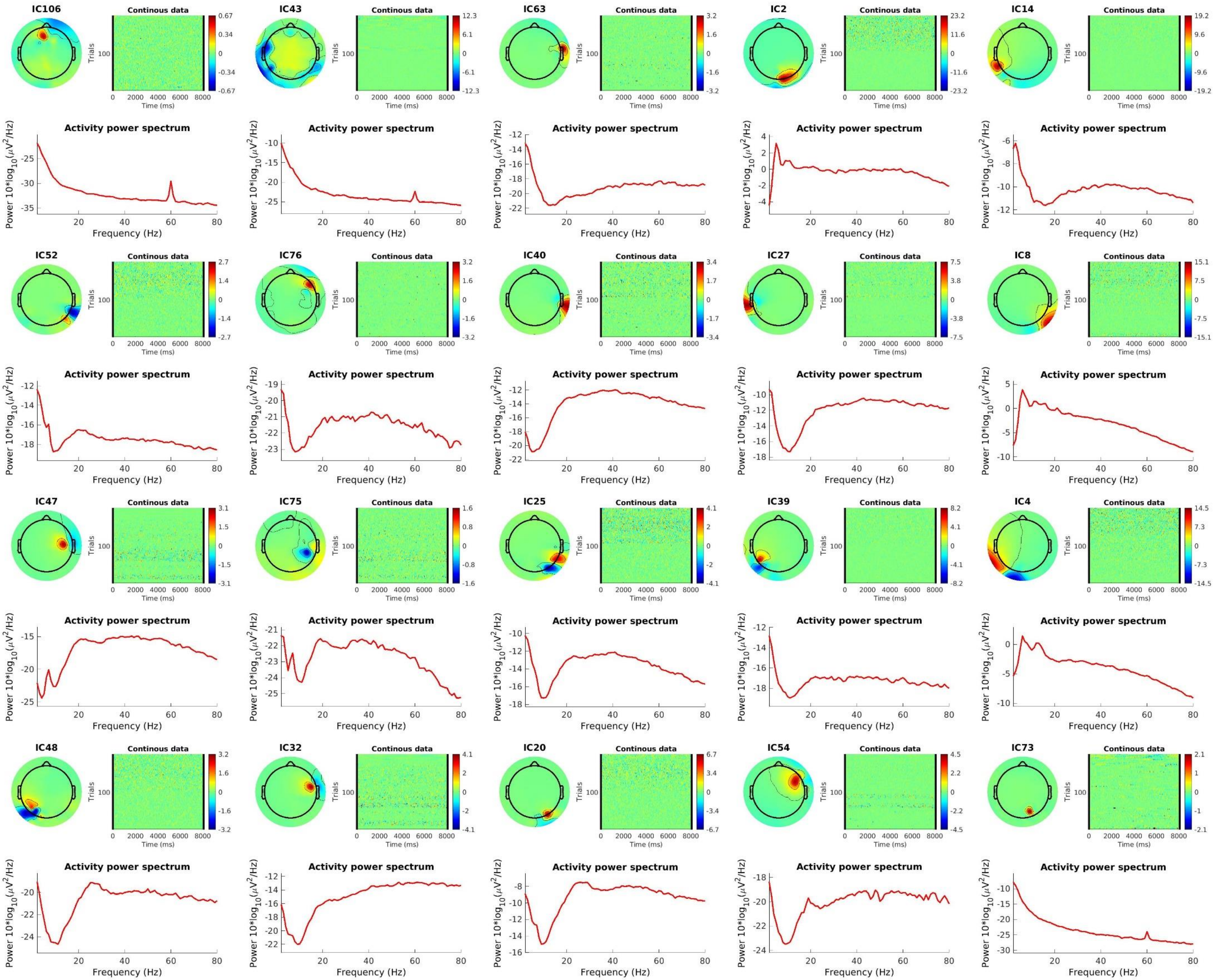
IC106
Continous data
Trials
Time (ms)
Activity power spectrum
Power 10*log$_{10}$($\mu$V$^2$/Hz)
Frequency (Hz)
IC43
IC63
IC2
IC14
IC52
IC76
IC40
IC27
IC8
IC47
IC75
IC25
IC39
IC4
IC48
IC32
IC20
IC54
IC73

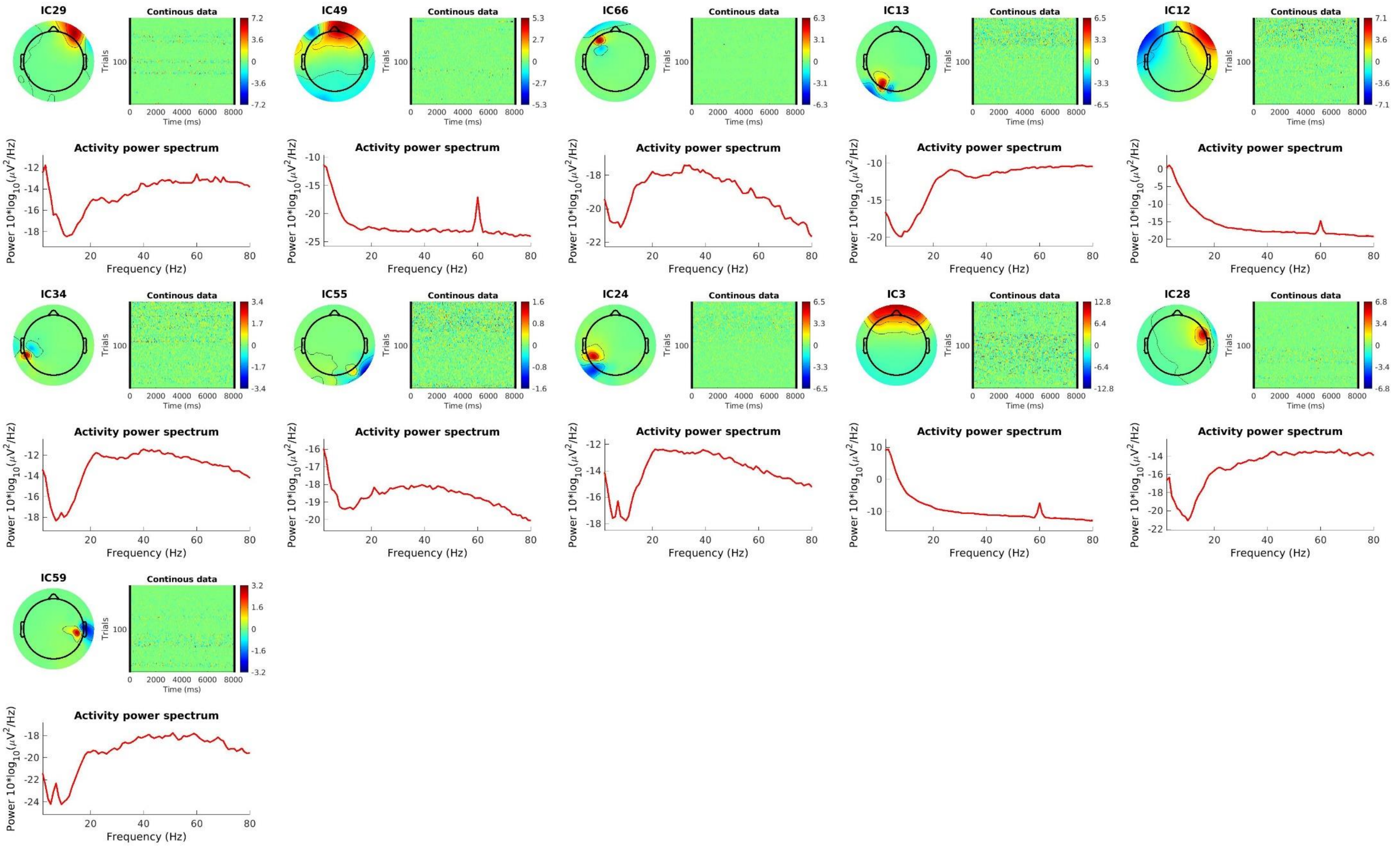
IC29
Continous data
Activity power spectrum
IC49
Continous data
Activity power spectrum
IC66
Continous data
Activity power spectrum
IC13
Continous data
Activity power spectrum
IC12
Continous data
Activity power spectrum
IC34
Continous data
Activity power spectrum
IC55
Continous data
Activity power spectrum
IC24
Continous data
Activity power spectrum
IC3
Continous data
Activity power spectrum
IC28
Continous data
Activity power spectrum
IC59
Continous data
Activity power spectrum
Trials
Time (ms)
Frequency (Hz)
Power 10*log10(μV2/Hz)

## Low Functioning Older Adult Without iCanClean

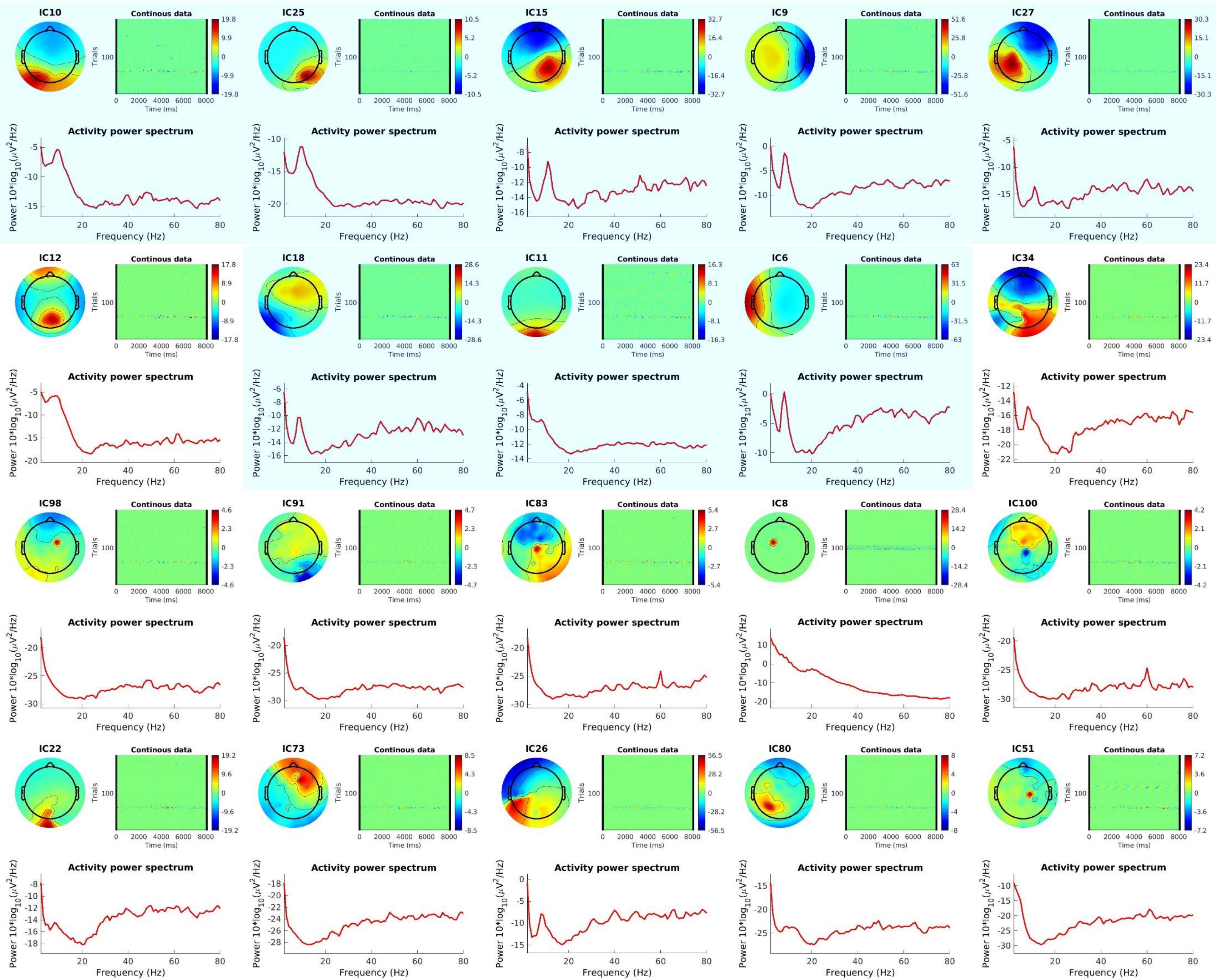

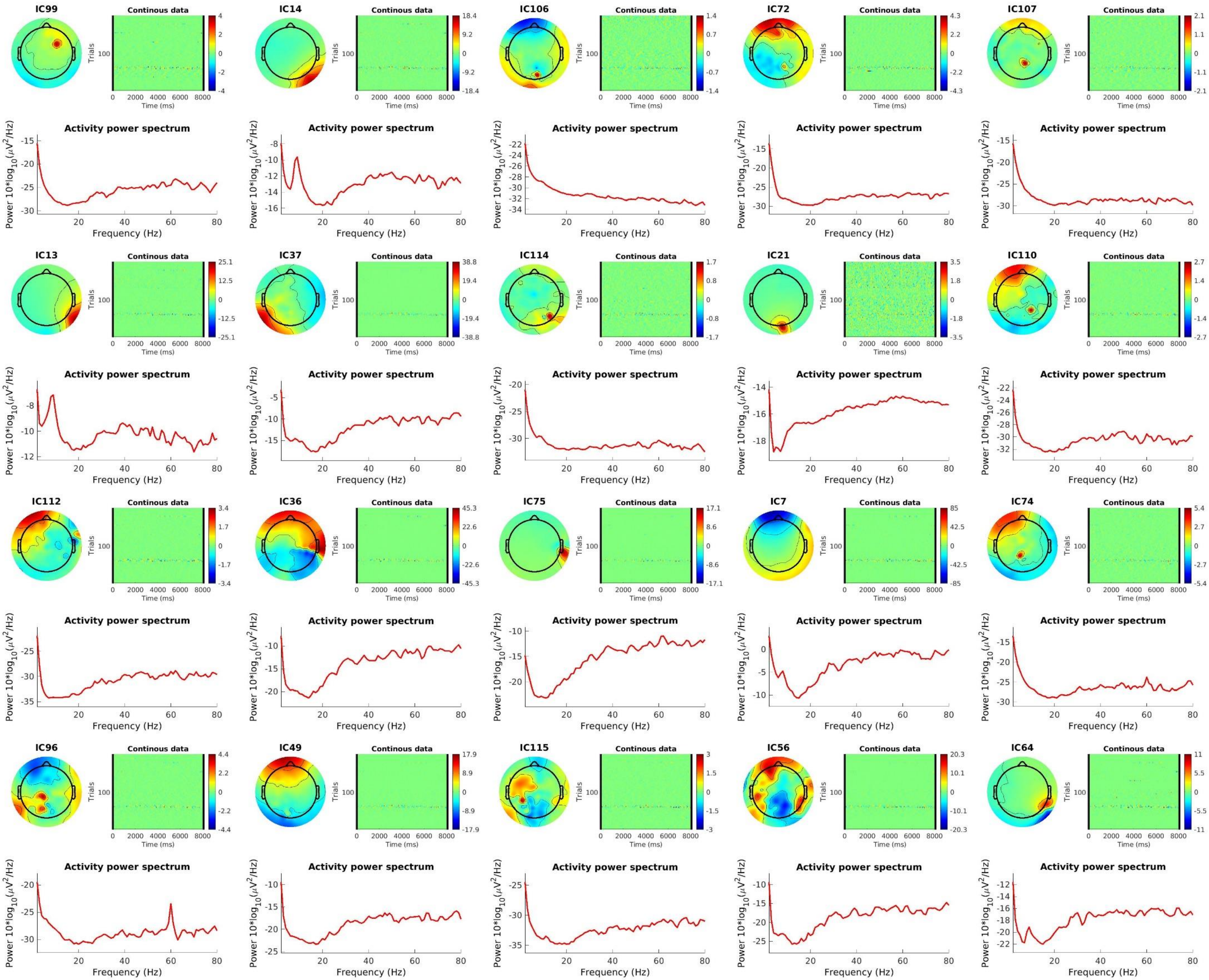
IC99
IC14
IC106
IC72
IC107
IC13
IC37
IC114
IC21
IC110
IC112
IC36
IC75
IC7
IC74
IC96
IC49
IC115
IC56
IC64
Continous data
Trials
Time (ms)
Activity power spectrum
Power 10*log10(μV2/Hz)
Frequency (Hz)

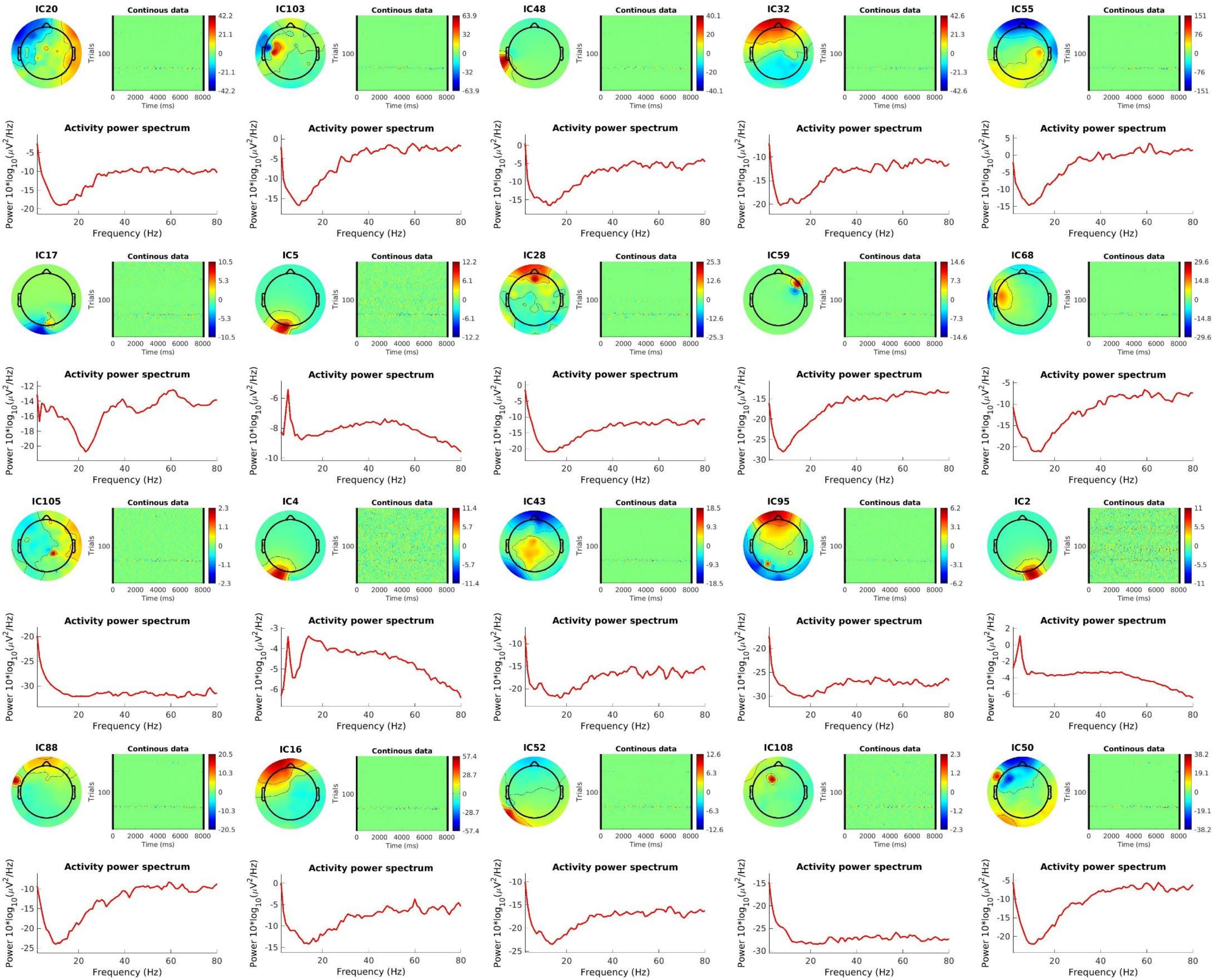
IC20
IC103
IC48
IC32
IC55
IC17
IC5
IC28
IC59
IC68
IC105
IC4
IC43
IC95
IC2
IC88
IC16
IC52
IC108
IC50
Continous data
Trials
Time (ms)
Activity power spectrum
Power 10*log$_{10}$($\mu$V$^2$/Hz)
Frequency (Hz)

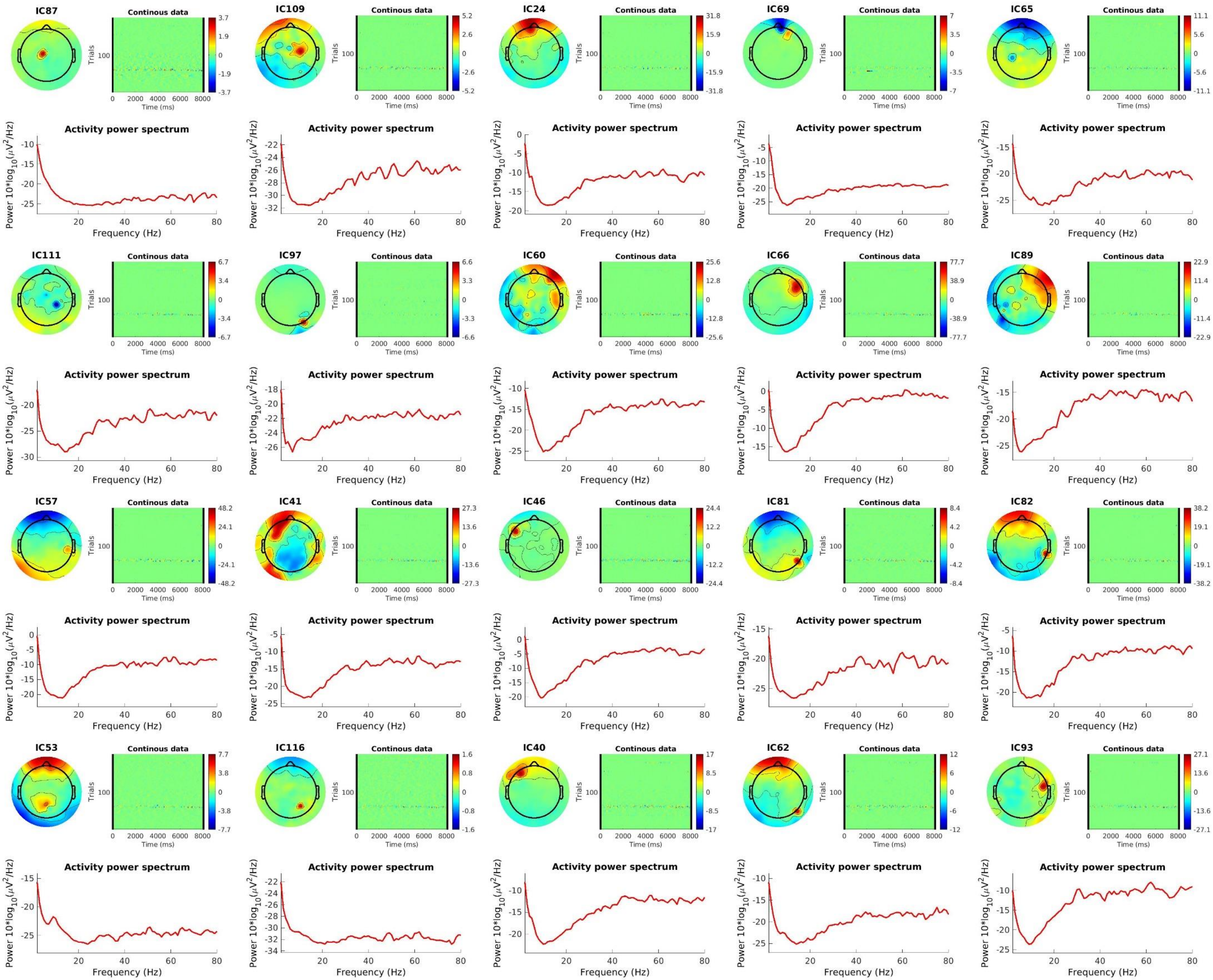
IC87
Continous data
Activity power spectrum
IC109
Continous data
Activity power spectrum
IC24
Continous data
Activity power spectrum
IC69
Continous data
Activity power spectrum
IC65
Continous data
Activity power spectrum
IC111
Continous data
Activity power spectrum
IC97
Continous data
Activity power spectrum
IC60
Continous data
Activity power spectrum
IC66
Continous data
Activity power spectrum
IC89
Continous data
Activity power spectrum
IC57
Continous data
Activity power spectrum
IC41
Continous data
Activity power spectrum
IC46
Continous data
Activity power spectrum
IC81
Continous data
Activity power spectrum
IC82
Continous data
Activity power spectrum
IC53
Continous data
Activity power spectrum
IC116
Continous data
Activity power spectrum
IC40
Continous data
Activity power spectrum
IC62
Continous data
Activity power spectrum
IC93
Continous data
Activity power spectrum
Trials
Time (ms)
Frequency (Hz)
Power 10*log10(μV2/Hz)

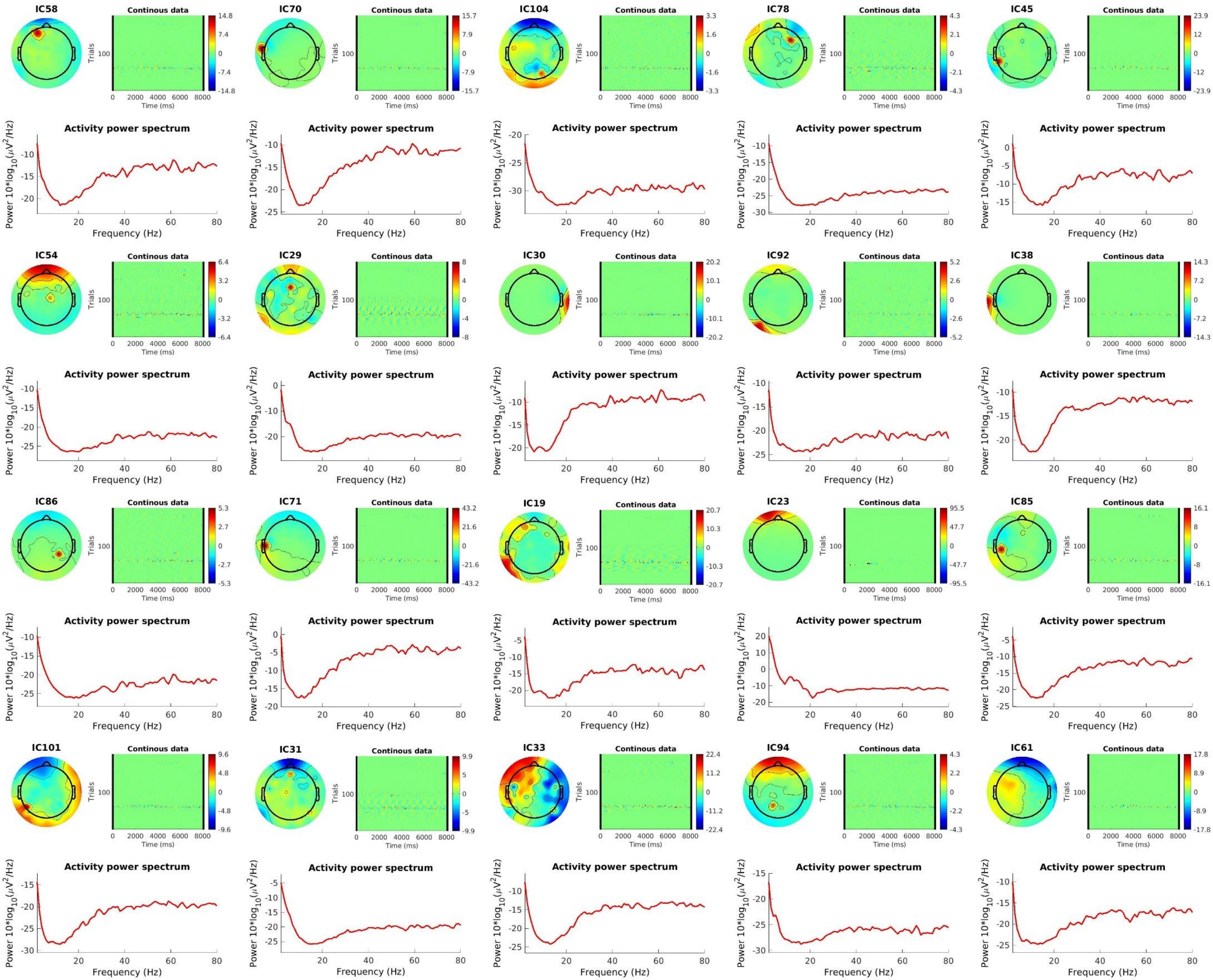
IC58
Continous data
Trials
Time (ms)
Activity power spectrum
Power 10*log10(μV2/Hz)
Frequency (Hz)
IC70
Continous data
Activity power spectrum
IC104
Continous data
Activity power spectrum
IC78
Continous data
Activity power spectrum
IC45
Continous data
Activity power spectrum
IC54
Continous data
Activity power spectrum
IC29
Continous data
Activity power spectrum
IC30
Continous data
Activity power spectrum
IC92
Continous data
Activity power spectrum
IC38
Continous data
Activity power spectrum
IC86
Continous data
Activity power spectrum
IC71
Continous data
Activity power spectrum
IC19
Continous data
Activity power spectrum
IC23
Continous data
Activity power spectrum
IC85
Continous data
Activity power spectrum
IC101
Continous data
Activity power spectrum
IC31
Continous data
Activity power spectrum
IC33
Continous data
Activity power spectrum
IC94
Continous data
Activity power spectrum
IC61
Continous data
Activity power spectrum

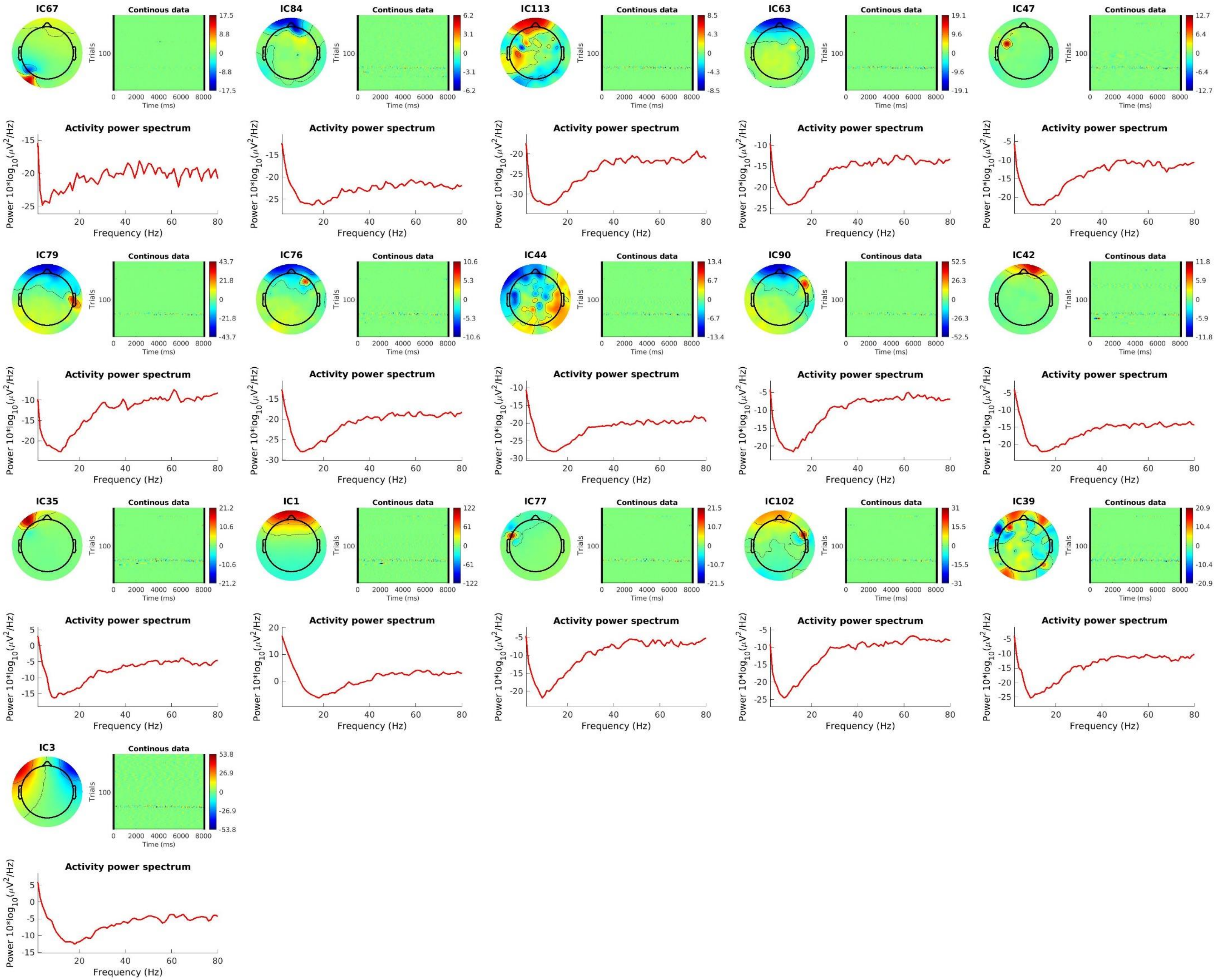

IC67
Continous data
17.5
8.8
0
-8.8
-17.5
IC84
6.2
3.1
-3.1
-6.2
IC113
8.5
4.3
-4.3
-8.5
IC63
19.1
9.6
-9.6
-19.1
IC47
12.7
6.4
-6.4
-12.7
IC79
43.7
21.8
-21.8
-43.7
IC76
10.6
5.3
-5.3
-10.6
IC44
13.4
6.7
-6.7
-13.4
IC90
52.5
26.3
-26.3
-52.5
IC42
11.8
5.9
-5.9
-11.8
IC35
21.2
10.6
-10.6
-21.2
IC1
122
61
-61
-122
IC77
21.5
10.7
-10.7
-21.5
IC102
31
15.5
-15.5
-31
IC39
20.9
10.4
-10.4
-20.9
IC3
53.8
26.9
-26.9
-53.8
Trials
100
0 2000 4000 6000 8000
Time (ms)
Activity power spectrum
Power 10*log$_{10}$($\mu$V$^2$/Hz)
Frequency (Hz)
20 40 60 80

# Low Functioning Older Adult With iCanClean

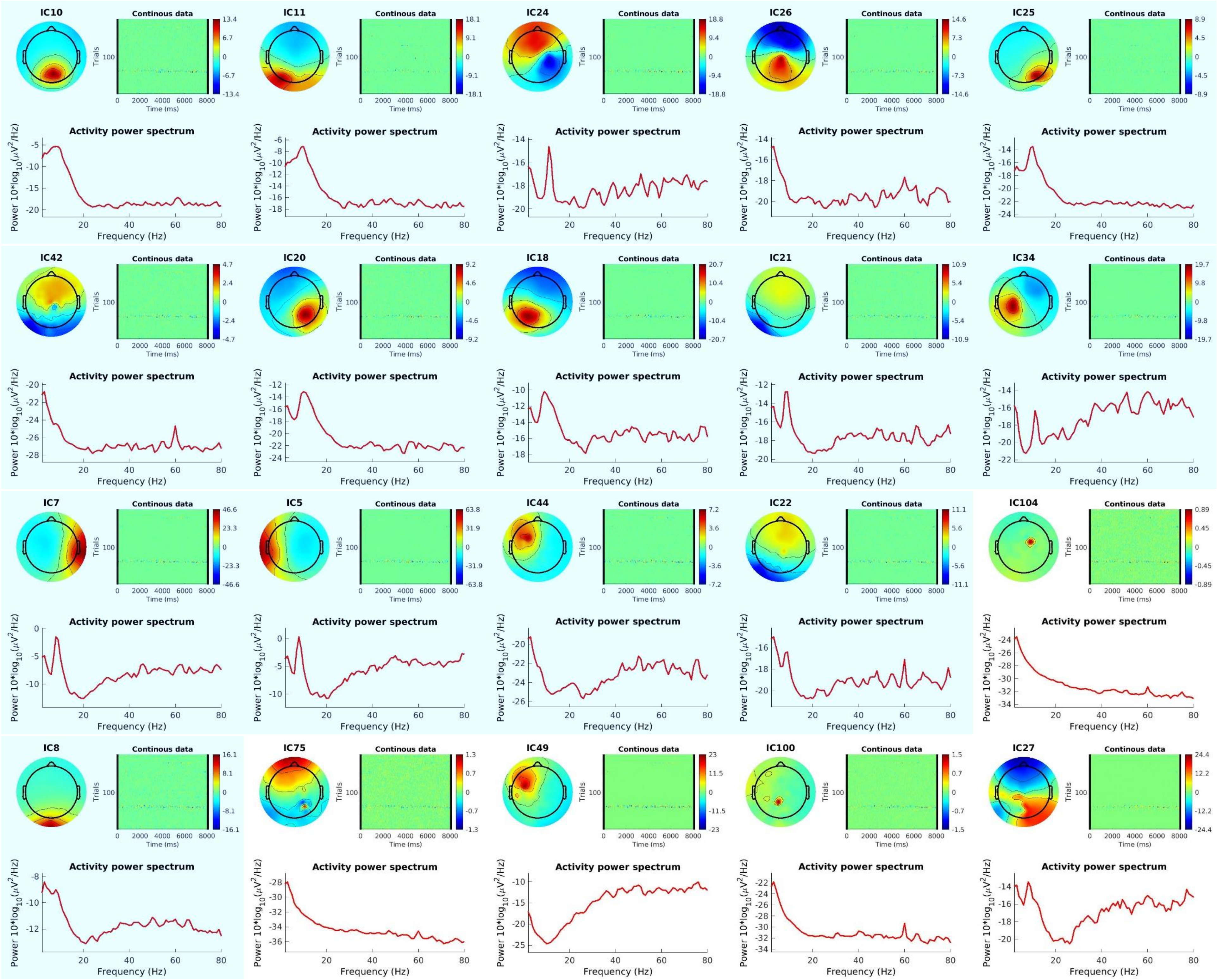

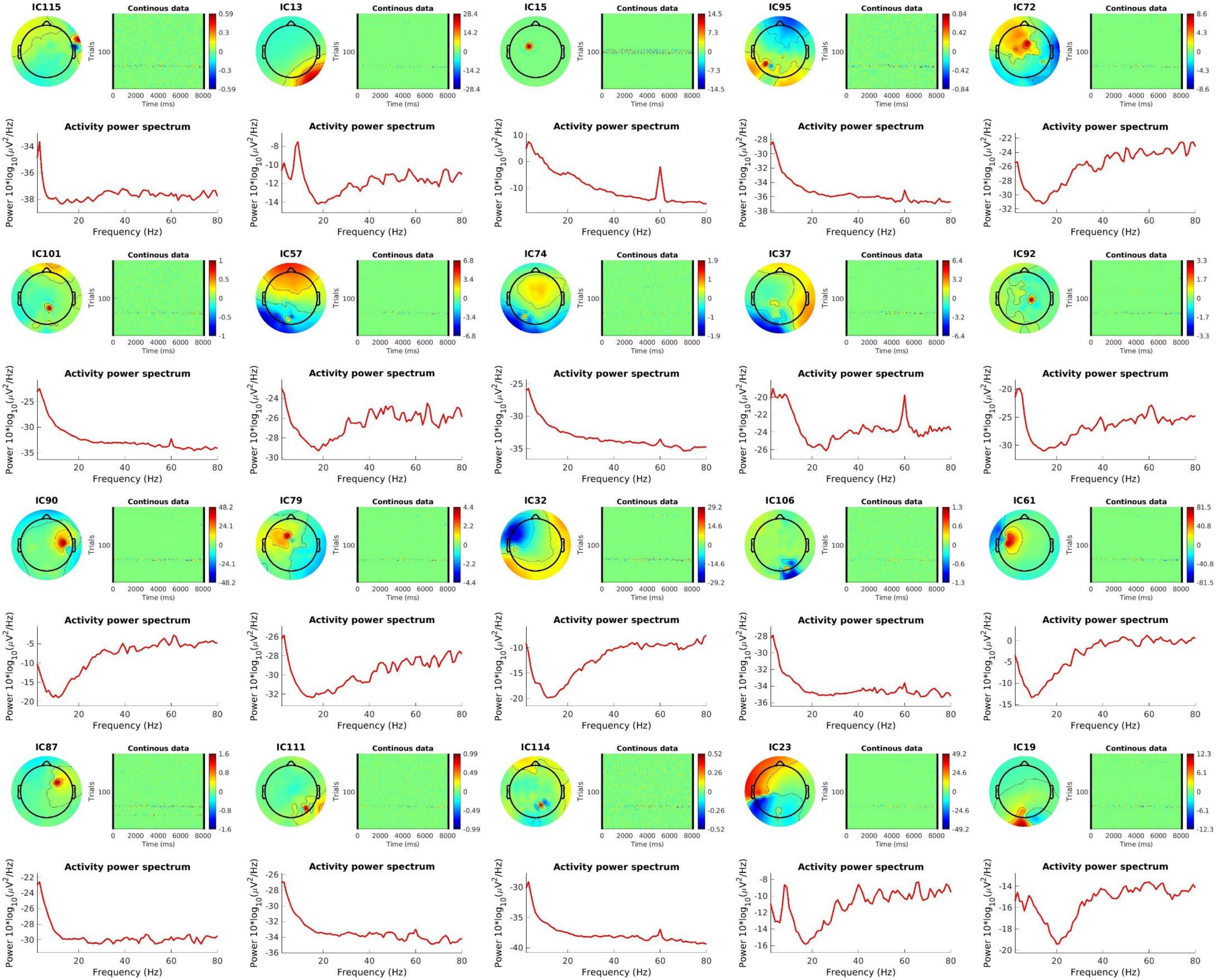
IC115
IC13
IC15
IC95
IC72
IC101
IC57
IC74
IC37
IC92
IC90
IC79
IC32
IC106
IC61
IC87
IC111
IC114
IC23
IC19
Continous data
Trials
Time (ms)
Activity power spectrum
Power 10*log10(μV2/Hz)
Frequency (Hz)

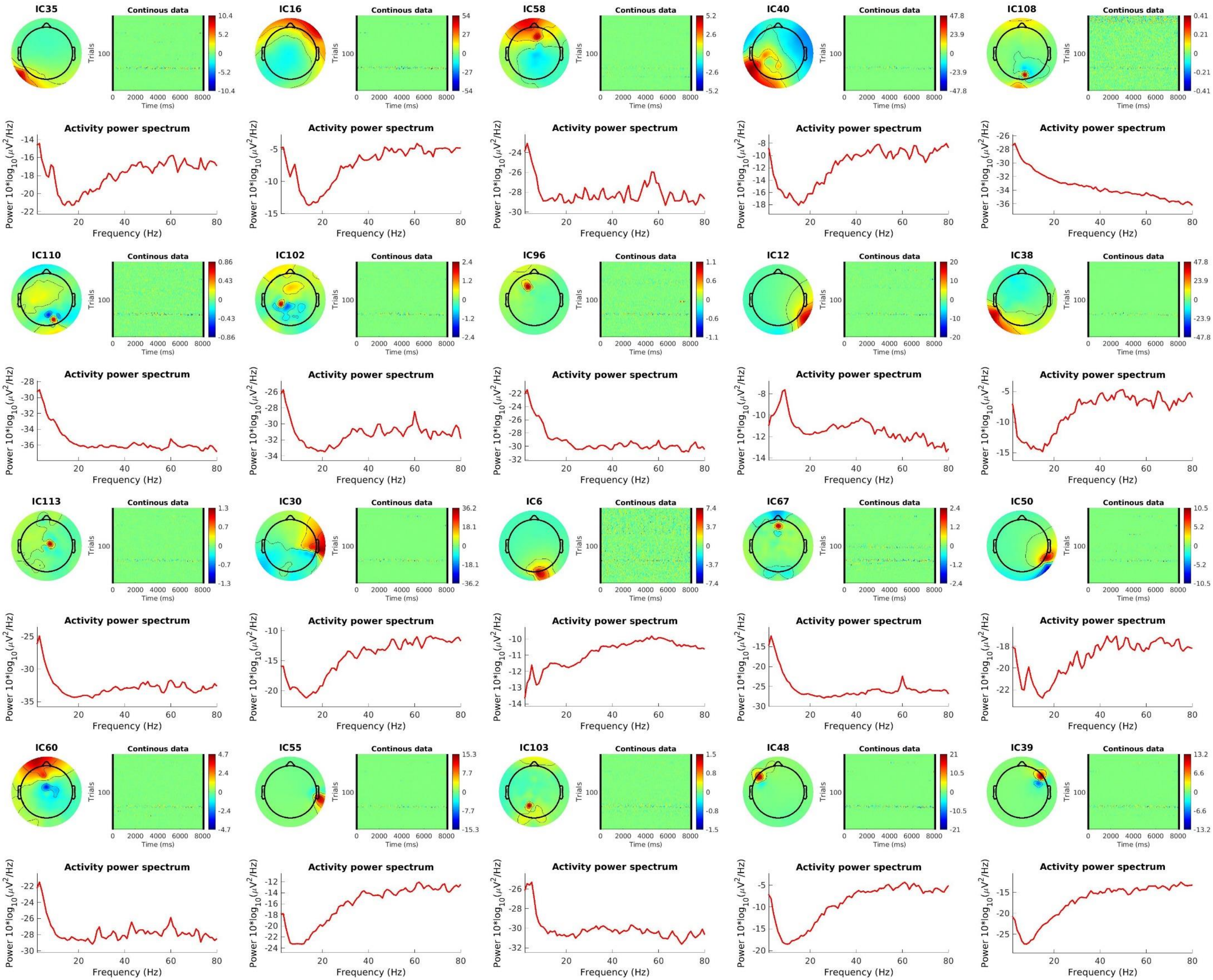
IC35
IC16
IC58
IC40
IC108
IC110
IC102
IC96
IC12
IC38
IC113
IC30
IC6
IC67
IC50
IC60
IC55
IC103
IC48
IC39
Continous data
Trials
Time (ms)
Activity power spectrum
Power 10*log$_{10}$($\mu$V$^2$/Hz)
Frequency (Hz)

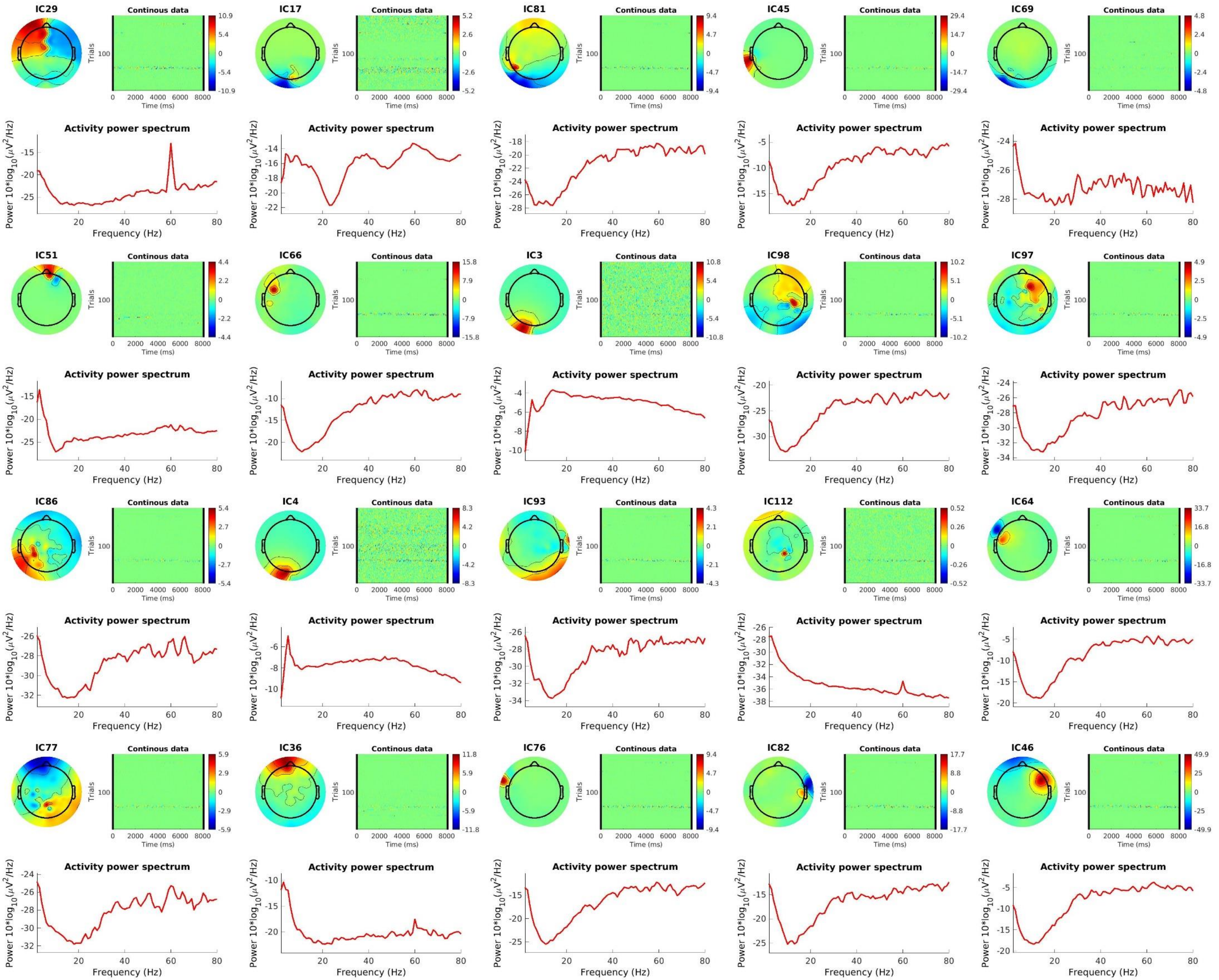
IC29
IC17
IC81
IC45
IC69
IC51
IC66
IC3
IC98
IC97
IC86
IC4
IC93
IC112
IC64
IC77
IC36
IC76
IC82
IC46
Continous data
Trials
Time (ms)
Activity power spectrum
Power 10*log10(μV2/Hz)
Frequency (Hz)

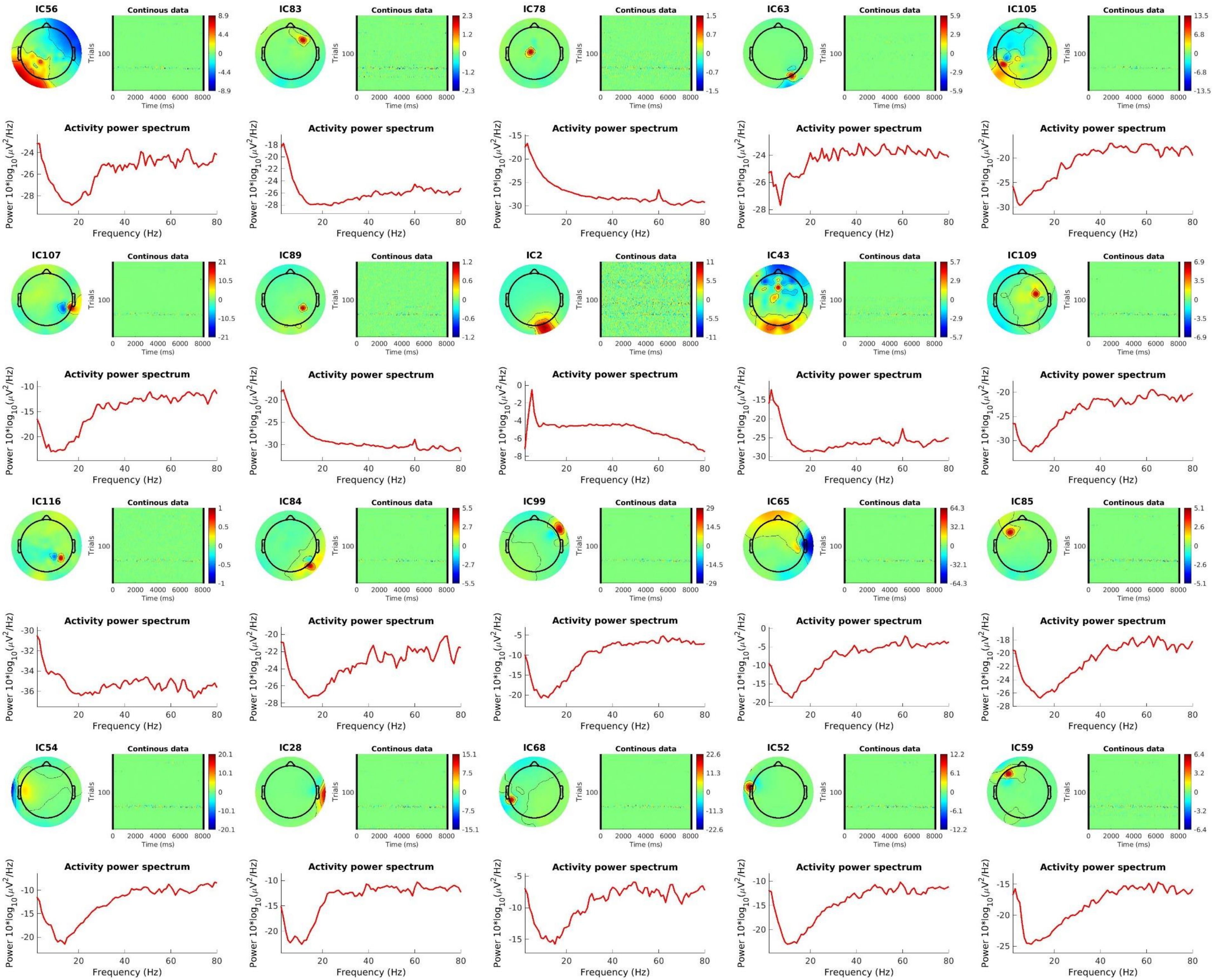
IC56
IC83
IC78
IC63
IC105
IC107
IC89
IC2
IC43
IC109
IC116
IC84
IC99
IC65
IC85
IC54
IC28
IC68
IC52
IC59
Continous data
Trials
Time (ms)
Activity power spectrum
Power 10*log10(μV²/Hz)
Frequency (Hz)

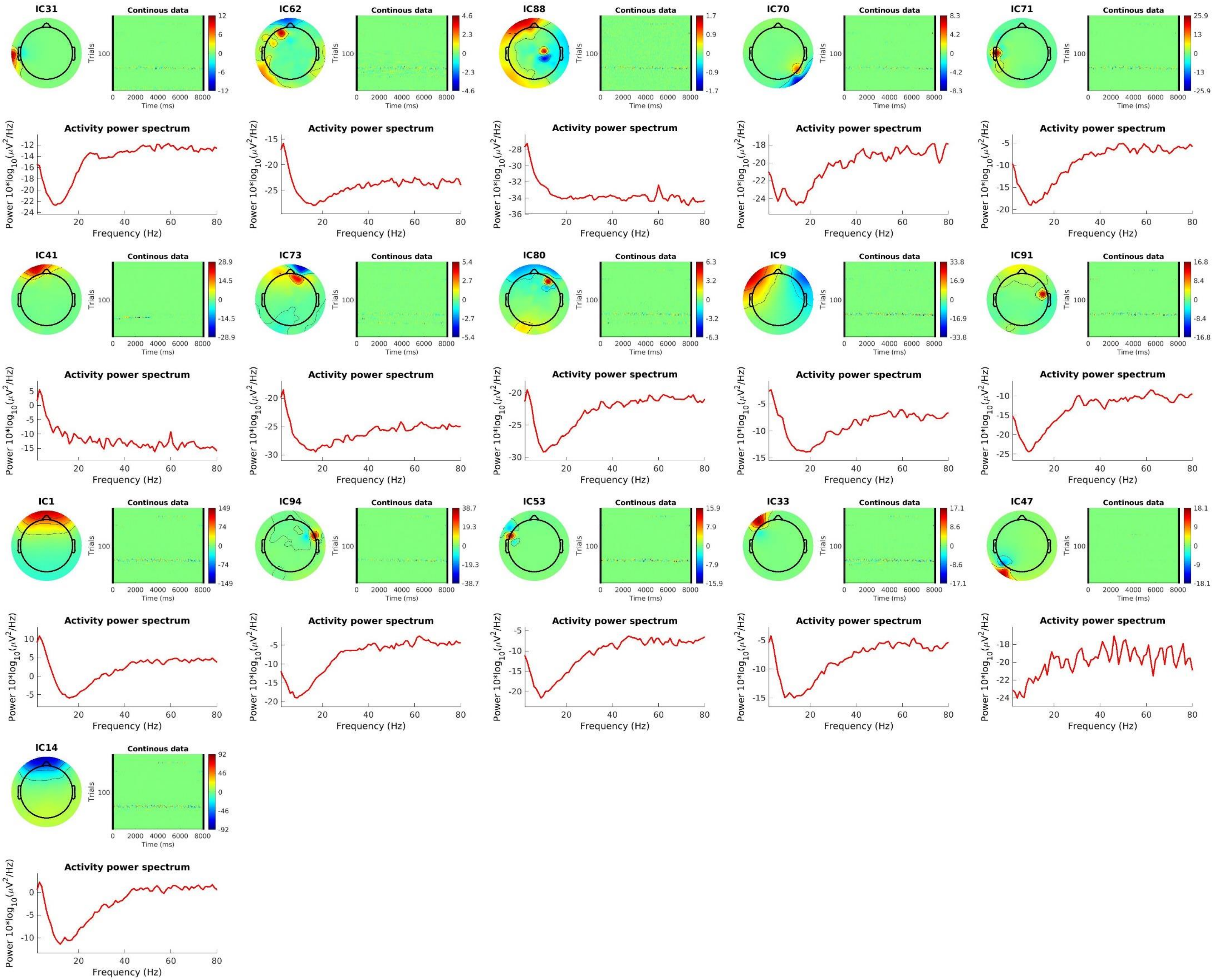
IC31
Continous data
Activity power spectrum
IC62
Continous data
Activity power spectrum
IC88
Continous data
Activity power spectrum
IC70
Continous data
Activity power spectrum
IC71
Continous data
Activity power spectrum
IC41
Continous data
Activity power spectrum
IC73
Continous data
Activity power spectrum
IC80
Continous data
Activity power spectrum
IC9
Continous data
Activity power spectrum
IC91
Continous data
Activity power spectrum
IC1
Continous data
Activity power spectrum
IC94
Continous data
Activity power spectrum
IC53
Continous data
Activity power spectrum
IC33
Continous data
Activity power spectrum
IC47
Continous data
Activity power spectrum
IC14
Continous data
Activity power spectrum
Trials
Time (ms)
Power $10*\log_{10}(\mu V^2/Hz)$
Frequency (Hz)
